# Pharmacotherapy of Patients Taking New Psychoactive Substances: A Systematic Review and Analysis of Case Reports

**DOI:** 10.3389/fpsyt.2021.669921

**Published:** 2021-04-23

**Authors:** Michal Ordak, Aleksandra Zmysłowska, Miłosz Bielski, Daniel Rybak, Maja Tomaszewska, Katarzyna Wyszomierska, Aleksandra Kmiec, Natalia Garlicka, Maria Zalewska, Michal Zalewski, Tadeusz Nasierowski, Elzbieta Muszynska, Magdalena Bujalska-Zadrozny

**Affiliations:** ^1^Department of Pharmacodynamics, Centre for Preclinical, Research and Technology (CePT), Medical University of Warsaw, Warsaw, Poland; ^2^Department of Psychiatry, Medical University of Warsaw, Warsaw, Poland; ^3^Department of Medical Biology, Medical University of Bialystok, Bialystok, Poland

**Keywords:** new psychoactive drugs, case report, psychopharmacology, pharmacotherapy, substances abuse

## Abstract

**Background:** In recent years, an increase in the frequency of hospitalizations of patients taking newer and newer psychoactive substances has been observed around the world. Each year, authors publish case reports of patients who consumed previously unknown NPS. Most publications of this type concern the period between 2014 and 2016. However, no publication systematically reviews the pharmacotherapy used in these cases. This study aims to review the case reports of patients taking NPS published between 2010 and 2019, as well as analyzing the pharmacotherapy used.

**Methods:** We searched the Thomson (Web of Knowledge), PubMed/Medline, Science Direct, Scopus and Google Scholar databases. The search was performed using all possible combinations of the term “case report” describing the use of NPS, also referred to as designer medications, internet medications, research chemicals and herbal highs.

**Results:** We analyzed 51 case reports on the intake of various types of NPS. Most of them (*p* < 0.001) concerned the use of synthetic cannabinoids (41.2%) and cathinones (31.4%). The pharmacotherapy applied primarily (*p* < 0.001) consisted of administering benzodiazepines to patients (62.7%), most of whom took only this group of medications (25.5%), followed by groups receiving benzodiazepines combined with neuroleptics (15.7%) and muscle relaxants (11.8%). Opioids were administered primarily to patients taking synthetic opioids (*p* < 0.001). Of the 5 cases of deaths from NPS reported in the literature, three relate specifically to the synthetic opioid MT-45. The later the time period, the more medications patients were administered (*p* = 0.02).

**Conclusion:** In the pharmacotherapy for NPS poisoning, one should focus primarily on combating psychomotor agitation.

## Introduction

In recent years, there has been a sharp increase in the frequency of hospital admissions of patients who take new psychoactive substances (NPS). The problem of NPSs has been observed in many countries around the world, where the substances are also known as “legal highs,” “designer drugs,” “herbal highs,” “spice,” or “research chemicals” (1).

A few thousand NPS were preliminarily identified in the “NPS.Finder®”, which is four times bigger number than the figure suggested by European and international drug agencies. Pharmacodynamics and pharmacokinetics of NPS are not fully understood due to their constantly evolving chemical composition (2).

In clinical practice, it is often difficult to determine the appropriate diagnostic methods and standards of treatment and management in emergencies caused by NPS. As we lack simple, specific tests to detect NPSs in patients' bodily fluids or tissues, there is, regrettably, no specific method of treatment for NPS poisoning. Another problem is the fact that patients combine NPSs with other psychoactive substances, which also often blurs the clinical picture (1, 3).

In the literature, there is no review describing previously published scientific papers on the pharmacotherapy of patients receiving various types of NPSs. The division was made into three time periods: until up to 2013, from 2014 to 2015, and from 2016 to 2019. This is due to the fact that the first publications appeared in the first of these periods, in which the authors included case reports of patients who had taken NPSs. Between 2014 and 2015, articles described the treatment used for many of the newer NPSs which had appeared on the market. Based on the 2014 data from the European Drug Report of the European Monitoring Center for Drugs and Drug Addiction (EMCDDA), a record 101 new psychoactive substances were detected in Europe (4). In 2016, the European Monitoring Center for Drugs and Drug Addiction (EMCDDA) drew attention to the constantly emerging new substances and the changing patterns of drug use. According to the 2016 EMCDDA report, the analysis highlighted the need to consider a number of more complex aspects of the problem in European drug strategy, including NPSs, compared to the previous time period (5).

Because the literature on the subject covers case reports, an additional goal was to conduct a review and analysis of these reported cases.

## Materials and Methods

International databases, including Thomson (Web of Knowledge), PubMed/Medline, Science Direct, Scopus and Google Scholar were searched for case reports on the use of various types of new psychoactive substances (NPS) published between 2010 and 2019. The variables used in the analysis included: year of publication, age, gender, NPS taken, clinical symptoms, pharmacological treatment applied and deaths. The search was performed using all possible combinations of the term “case report” describing the use of NPS, also referred to as designer medications, internet medications, research chemicals, and herbal highs. Additionally, when searching the databases, the abbreviations and full names of NPS were entered, such as MT-45 [1-cyclohexyl-4- (1,2-diphenylethyl) piperazine] and mephedrone [4-methyl methcathinone (4-MMC)].

### Pharmacotherapy of Patients Taking NPSs

#### 2010–2013

##### Synthetic Cathinones

Synthetic analogs of drugs such as methamphetamine and cocaine were legally sold as “bath salts” between 2010 and 2013. Synthetic cathinones are β-ketophenethylamines, which are structurally similar to amphetamines and are therefore abused. Cathinone, and to a lesser extent its metabolite—cathine—are responsible for the amphetamine-like euphoric effects, which are achieved by chewing the leaves and twigs of the khat plant (Catha edulis). Many psychoactive substances, including mephedrone, methylenedioxypyrovalerone (MDPV), methylone, butylone, and naphirone, have been identified in the newer products, so-called bath salts. Cathinone, mephedrone, methcathinone, and methylone have been shown to strongly inhibit the reuptake of dopamine, serotonin, and noradrenaline. These substances also increase the presynaptic release of the same monoamines, but to a lesser extent (6, 7). The literature offers limited data on the pharmacokinetics and pharmacodynamics of synthetic cathinones in humans.

Penders and Gestring (8) describe the case of three people who consumed bath salts. The patients suffered from hallucinations, were hyperactive and aggressive, and had insomnia. Two of them were treated with risperidone orally at a dosage of 0.5 mg twice daily. One of the men was given 1 mg of haloperidol. After 2–3 days of therapy, the symptoms of paranoia subsided (8).

Antonowicz et al. (6) describe the case of a 27-year-old girl and her 32-year-old boyfriend who were brought to a hospital after reporting to the police that they were under attack. Police officers found them barricaded in the bedroom, claiming that there was a corpse in the hallway and that they had been killed. Upon reaching the emergency room, they had tachycardia, were scared, and were perspiring profusely. Blood and urine counts were normal. They were transferred to a psychiatric ward where they were monitored. The woman was given 0.5 mg of risperidone; the man was not administered any antipsychotic drugs during his hospital stay. The patients admitted that they had been consuming an illegally obtained drug, Suboxone. Then they started taking a product called “Powdered Rush.” They were ingesting it by inhalation for 5–6 days before being admitted to the hospital. The substance ingested by the couple contained MDPV and was marketed as “bath salts” (6).

Mangewala et al. (9) cite the case of a 15-year-old boy with no prior psychiatric history. He reported to the emergency room complaining of agitation and psychotic symptoms. The patient had reportedly smoked marijuana laced with bath salts. Soon afterwards, the patient became paranoid and barricaded himself in his father's house. The police were called to forcibly enter the house and the patient was taken to the emergency room. The patient's agitation continued to worsen and laboratory tests revealed an elevated level of creatine phosphokinase (CPK). The urine toxicology report was negative. The patient was treated in a hospital intensive care unit (ICU) where he continued to exhibit psychotic and agitated behavior, at one point attacking a staff member. He returned home a few days later once his health had stabilized, although at home he was still showing signs of paranoia. When the patient presented again with symptoms of paranoia a month later, his father took him back to the hospital, where he was referred for psychiatric hospitalization. During the first interview, during which he was non-verbal, the patient was diagnosed with periods of extreme psychomotor impairment. In another situation, he seemed confused, and kept repeating questions to himself. He made paranoid statements such as, “Don't let them take me!” and “How do I get out of this?” The patient reported that nothing around him was real. He claimed that his father had been replaced by an impostor, and that his sister had been hurt by unknown parties. During the interview, he was able to admit that some part of his paranoia was not real. He reported fear but denied any thoughts, intentions, or plans that were suicidal or homicidal in nature. The patient's family was also interviewed and denied that the patient had any history of psychiatric problems or similar behavior. In addition, they reported no history of head injuries or epileptic seizures and no family history of psychiatric illness. However, they declared that the patient had used marijuana in the past without any negative side effects. As mentioned earlier, the patient stated that, to his belief, he was smoking marijuana mixed with “bath salts.” While hospitalized, the patient was treated with a combination of 5 mg of olanzapine once daily and 0.5 mg of lorazepam twice daily. The dose of olanzapine was increased to 7.5 mg per day, which combined with the lorazepam reduced the symptoms of psychosis and agitation. He began to interact with his peers. The symptoms of paranoia resolved within 3 days of treatment and the patient was discharged with a prescription for 7.5 mg of olanzapine once daily, 0.5 mg of lorazepam twice daily, and outpatient follow-up. He had no recurrence of symptoms during the 8-week follow-up period. He denied continued use of the substance. No further adjustments in dosage were required (9).

Lenz et al. (10) report the story of a 22-year-old man who found himself in the Emergency Department (ED) after ingesting 1 g of a substance which he called Cristalius the previous night. Mephedrone and a synthetic cathinone derivatives were presumed as the main ingredients.

That morning, after running three miles to start his intense physical exercise, he felt dizzy and light-headed. He was transported to his military aid station and had a brief syncopal episode on the way. He woke up at the aid station confused and belligerent, and therefore had to be handcuffed. Eventually, he calmed down enough to be taken to the hospital, but had a tachycardia of up to 173 beats per minute during the transport. After arriving at the ED, he was given 2 mg of lorazepam for sedation. He remained confused. His heart rate slowly returned to normal, and within 3 h it had dropped to 100 beats per minute with a blood pressure of 129/50 mmHg. He was aware of people, his surroundings, and the time and was less anxious, though he complained of fatigue. The clinically significant laboratory results at that time were a creatine kinase level of 668 U/l (norm: 25–200), a creatinine level of 1.35 mg/dl (norm: 0.66–1.25), and a troponin level of 0.516 ng/ml (norm: 0.0–0.034). One week prior to his admission to the hospital, the patient's urine screening test was positive for amphetamines. He claimed it was because he had borrowed Adderall from a friend, but tested negative for the drug while hospitalized. Intravenous hydration was also initiated; his creatinine levels dropped to 0.97 and his creatine kinase levels rose to 1544. The patient, however, remained asymptomatic with no muscle pain. He only confessed to using marijuana in high school and denied taking any other illegal substances before using Cristalius twice. He denied any past or current symptoms of mood disorders or post-traumatic stress. He refused a positive drug test on his ward and stated that he consumed his last alcoholic drink 6–7 months earlier. At the time of discharge, the patient was stable and asymptomatic (10).

Nervous system stimulants, including methamphetamine and cocaine, are responsible for permanent changes in the dopaminergic reward system in the brain. This causes persistent dysphoria after drug withdrawal (11). There are also psychoactive substances that produce a psychotic syndrome similar to schizophrenia. Usually, these symptoms disappear with treatment, but a few cases of persistent hallucinations have been reported after stopping amphetamine use. Electroshock has been shown to alleviate these types of hallucinations. Synthetic cathinones, which are also stimulants, cause similar symptoms as the above-mentioned products. The case of a patient who was abusing MDPV for several months has been described. He suffered from persistent symptoms of psychosis, visual hallucinations, suspicion, and social withdrawal. After unsuccessful attempts at antipsychotic treatment, electroconvulsive therapy was performed (12).

Penders et al. (12) report the case of a 26-year-old woman who suffered from severe hallucinations and social withdrawal. These symptoms had appeared 8 months earlier, after she had taken bath salts, and persisted even after the substance was no longer being consumed. The patient had been using these substances for 13 months before being admitted to the hospital, but the first hallucinations began 5 months later. There were no serious mental disorders in her family medical history. No improvement was observed under prior treatment with haloperidol and risperidone; minimal improvement was noted only after the administration of 120 mg of lurasidone nightly. The patient was also prescribed 40 mg of citalopram and 300 mg of trazodone for sleep. Before commencing a modified bilateral electroconvulsive therapy, the patient was subjected to general anesthesia consisting of intravenous injection of 100 mg of methohexital and 100 mg of succinylcholine. The Somatic Thymatron IV device was used at the short heart rate setting, the stimulus being 15% (75.6 milicouloms or 15 J). The initial dosage of 120 mg of lurasidone was still being administered at nighttime. After two treatments with a seizure time of 31 and 44 s, the patient reported a reduction in visual hallucinations. Lurasidone was then discontinued. After two more treatments with a seizure time of 19 and 25 s, the hallucinations decreased again. The patient noticed an improvement in mood and a reduction in social anxiety, after which she was discharged from the hospital. Eight months after the end of treatment, the woman continued to experience occasional hallucinations, fewer psychotic symptoms with improved social functions, and a complete disappearance of the previous suspicions. This was the first case to be described in which the symptoms of depression and hallucinations persisted after cessation of synthetic cathinone use (bath salts). In this case, the patient's condition did not improve on antipsychotics and antidepressants. A significant yet incomplete improvement was achieved after a short cycle of four standard electroconvulsive therapy sessions. During the abuse of stimulants, including synthetic cathinones, the level of dopamine changes. Some evidence suggests that generalized seizures lead to the release of monoamines, which confirms the beneficial effects of electroconvulsive shock therapy in schizophrenia. Electroshock therapy and antipsychotics have also been shown to work synergistically. Electroconvulsive therapy may be beneficial in patients with persistent psychotic symptoms following drug abuse, including synthetic cathinones (12).

Another patient with depressive disorders due to cathinone dependence was prescribed bupropion with a gradual increase in dosage to 450 mg per day, which along with the therapy sessions, helped to end these symptoms (13).

Lev-Ran et al. (13) present the case of a 23-year-old man. As a child, he was diagnosed with ADHD and was treated with methylphenidate for 3 years. Two years before the event in question, he started having depressive states and suicidal thoughts. During this time, he occasionally used cannabis and MDMA. Then he started taking cathinone daily. He claims that he was initially euphoric after consuming the substance, but within a few weeks the level of euphoria it caused decreased despite higher dosages. He gradually increased the daily intake, and swallowed up to 15 capsules (200 mg/capsule) per day. He worked during this time and was not intoxicated during working hours. His regimen of use was to consume cathinone continuously after returning home in the afternoon until late at night, when he fell asleep exhausted. He reported regular effects of withdrawal-like dysphoria and agitation during working hours that resolved after returning home and consuming cathinone. After a few months, the effect of cathinone weakened and he began to inhale the contents of the capsules. In the following year, his cathinone use increased and his level of functioning gradually worsened. He reported a severe, constant dysphoric mood that would improve for a very short time after consuming cathinone. He took cathinone almost exclusively when he was alone and gradually withdrew from social activities. Eventually, he underwent a psychiatric evaluation. His symptoms and complaints met the DSM-IV criteria for substance (amphetamine) addiction and major depression. After the initial evaluation, the patient agreed to weekly treatment sessions and was prescribed bupropion, with the dose gradually increasing to 300 mg/day. At the end of the meeting, the patient rated his motivation to stop cathinone treatment as “five out of ten” and confidence in abstaining from cathinone as “one in ten.” After 4 weeks of bupropion treatment, he reported a partial improvement in his mood as well as a significant reduction in cathinone cravings. He also reported reducing the frequency of cathinone use to twice a week and decreasing the amount to 5–10 capsules each time. At that time, he rated his confidence level of abstaining from cathinone as “five out of ten” and decided to return to ingesting (instead of inhaling) cathinone as part of a harm reduction strategy. The bupropion was increased to 450 mg/day and the weekly therapy continued for an additional 4 weeks. At the end of this period, the patient reported complete cathinone abstinence and markedly improved mood, and rated his confidence level in cathinone abstention as “nine out of ten.” He continued to abstain from cathinone and since remained in remission from depression for 12 months (13).

Garrett et al. (14) write about a 22-year-old man who presented to the ED. During the examination, the patient suffered from tachycardia and was sweating profusely. His pupils were dilated and he had a tremor at rest. The patient was unable to stand on his own. He had no drug allergies that he was aware of. He was taking fluoxetine (40 mg) and olanzapine (10 mg). He was also administered zopiclone at night. The patient had suffered from depression, mania, and self-harm for 2 years. Two bags were found on his person: a plastic bag labeled “Vegetable Food” and a brown paper bag labeled “Red Doves.” The patient admitted to taking approximately 40 capsules from these bag within a 4-h span; the main substance ingested was mephedrone. Adequate treatment of his serotonin syndrome (general supportive measures, IV fluids, and oral diazepam) led to the neurological symptoms resolving and the patient being discharged after 15 h (14).

Kasick et al. (15) report the case of a 38-year-old white male with no prior history of psychosis who claimed to have seen snakes and was acting oddly. He was given 2 mg of naloxone twice at the scene, but no improvement was observed. After 45 min of verbal de-escalation by the doctors, the patient was confined to an emergency bed and taken to an external hospital. While in the ED, he had tachycardia (the ECG revealed a pulse of 144, sporadic premature ventricular contractions (PVCs), and a QTc of 430 ms) and his temperature was 38.2°C. He was restrained on the bed, but he did not want to be covered with a blanket for fear of being bitten by “scorpions.” The patient had ingested bath salts packaged under the name “Arctic Blast” (a street name for synthetic cathinone) twice in the previous 2 days. He remained agitated in the emergency room and received 3 mg of intravenous lorazepam over 4 h with 2 L of IV fluids. Laboratory assays showed elevated levels of hemoglobin and hematocrit, consistent with his history of polycythemia. The white blood cell and platelet counts were normal. His first urine drug test was ostensibly only positive for phencyclidine (PCP); his serum ethanol level was zero. He remained very agitated and oversensitive and was given 4 mg of intravenous lorazepam on account of his attempts to leave the hospital. He was anxious and paranoid: he believed that people were stealing his possessions. He fought physically with security guards. Owing to his physical aggression, he was given a total of 6 mg of intravenous lorazepam and 5 mg of haloperidol. His creatine kinase (CK) level was elevated, at 1,468 IU/L. He was given IV fluids at 200 ml/ hour for 24 h. The additional drug screening done 9 h after the initial screening was positive for benzodiazepines, but negative for PCP or other substances. As time went on, the patient became calmer, but still had difficulties with awareness, orientation, cognitive speed, and attention. The next morning, 35 h after the patient's first exposure to emergency services, he remained alert and irritable, but his awareness had improved significantly. The patient clearly described how he had bought the “bath salts” from the local “head shop” and how he had drunk about half a spoonful of the bath salts in a carbonated cola drink twice in the 2 days prior to admission. He still vehemently denied ever using phencyclidine, although he did describe a more distant history of alcohol and cannabis use. He denied any history of psychosis or previous psychiatric treatment. His vital signs and increased CK levels returned to normal. Over the next 24 h, the patient showed a lasting improvement in attention, awareness, focus, concentration, and speed of thought. He had no residual psychotic symptoms and behaved normally on further cognitive screening. The next day, he was discharged (15).

Benzer et al. (16) report the case of a 36-year-old man with a history of alcohol and drug abuse who was admitted to hospital because of severe agitation and paranoia. The patient's girlfriend reported that the patient had been sober for ~20 months until he lost his job. Three days prior to admission, the patient began drinking alcohol and taking bath salts intranasally after sleepless nights. The night before taking the psychoactive substance, agitation, and auditory and visual hallucinations occurred; as a consequence, the patient believed that people were trying to harm him. On the morning of admission, shortly after taking more bath salts, he ran outside without any clothes on, shouting that someone was trying to strangle him. His girlfriend called the police, who found the patient running naked in the street. When the paramedics arrived, they found him being restrained by police officers, belligerent and confused, with illogical, paranoid, and rambling speech. His pulse was 157 beats per minute, with limited radial pulses, and his respiratory rate was 24 breaths per minute. His pupils were 5 mm in diameter. The patient was transported to the emergency room. On the way, he suddenly fell silent, and the paramedics suspected a seizure. They tried to administer midazolam but the patient removed the intravenous catheter. After arriving at the hospital, it was impossible to communicate with the patient. His history was obtained from his girlfriend. He had a history of depression and alcohol and drug abuse (including heroin, cocaine, and prescription opiates). His only medication was fluoxetine, which he reportedly had not taken for 2 weeks. During the examination, the patient was agitated, flailing his arms and legs, jerking his head, and making loud, incomprehensible sounds. He was unable to cooperate during the investigation and had to be restrained by several security officers. His pupils were equal and reactive to light; his gaze was focused upwards with slow, horizontal movements of the eyes. The patient's speech was quick and mostly incomprehensible, but he mentioned attacking and being attacked by animals, humans, and monsters. The rest of the examination proceeded normally. The patient was immobilized and administered intravenous midazolam followed by lorazepam, but his condition did not improve. Then, etomidate and rocuronium were administered, the trachea was intubated, and mechanical ventilation was begun, followed by sedation with propofol. A urinary catheter and an esophagus tube were inserted. In the next stage, fomepizole, sodium thiosulfate, sodium bicarbonate, saline solution, and potassium chloride were administered intravenously, after which he was admitted to the ICU and his condition improved (16).

##### Synthetic Cannabinoids

Synthetic cannabinoids first appeared in Europe in the mid-2000s and have been increasingly popular ever since. They are available on the Internet and in drug stores specializing in illegal drug accessories. These products contain several chemicals, including JWH018 and JWH-073 (naphthoylindoles), which have a similar mechanism to THC but appear to cause additional symptoms. “Spice” products contain several ingredients that are potent agonists of the cannabinoid receptors (17). Spice was initially the name of one of the popular brands, then became a generic term for other synthetic cannabinoid products, such as Black Mamba, Funky Monkey, K2, Popeye, Demon Smoke, Purple Haze, Dank, Hawaiian Harvest, and Vanilla Sky (18).

The effects of using synthetic cannabinoids vary from patient to patient, and include drowsiness, agitation, and odd behavior. Similar symptoms can present after marijuana use. Patients have reported somnolence, agitation, or paranoia. Other symptoms include tachycardia, warm and dry skin, and dilated pupils. These symptoms may suggest an anticholinergic or sympathomimetic effect. Lactic acidosis and leukocytosis have occurred, which may be due to agitation, respiratory failure, or possible seizure activity (18).

Data in the literature on patients who have overdosed on synthetic cannabinoids known as “Spice” show that lorazepam is effective at a dose of 4 mg to inhibit uncontrolled movement (19).

Simmons et al. (17) describe the case of a 25-year-old man who was admitted to the hospital due to two episodes which lasted several minutes. His blood pressure was 109/47 mmHg, his pulse was 122 beats per minute, his temperature was 37.3°C, and his glucose level was normal. The patient spontaneously moved his limbs to protect his airways and opened his eyes, but he did not respond to stimuli. His pupils were dilated with a slow reaction and intermittent esotropia. His skin was dry and warm. The electrocardiogram showed sinus tachycardia with a right bundle branch block pattern. No drugs were found in the urine and no alcohol was detected in the blood. The man did not use any drugs or alcohol, but 45 min before the episode he smoked a product called “Spice” for the first time. Laboratory analysis of his urine for synthetic cannabinoids was positive for JWH-018 metabolites and negative for JWH-073. After rehydration, the lactate level was 1.2 mmol/L. The patient was administered 4 mg of lorazepam and saline intravenously. After 3 h of observation, the patient's condition improved and the tachycardia resolved (17).

Kasick et al. (15) cite the case of a 36-year-old woman who was abusing illegal substances: synthetic cannabinoid preparations with the trade name “Black Mamba Spice.” The patient suffered from depression and migraine. She had a generalized tonic–clonic seizure that progressed into an epileptic seizure. The convulsions continued until she was re-intubated at the ED because she had been inadvertently intubated with an endotracheal tube in the esophagus. She was given lorazepam, etomidate, vecuronium, propofol, levetiracetam, and phenytoin. Laboratory tests for alcohol, THC, opiates, amphetamines, and cocaine were negative. The WBC count was 1,2400/μl (abnormal), though the level normalized within 48 h. The patient did not have an elevated temperature and had a normal chest X-ray; an MRI of the brain without contrast was normal. The EEG was mildly encephalopathic, with no focal features or epileptiform discharges. The woman was extubated 12 h after admission. She admitted that she had smoked Black Mamba Spice for the first time before the seizure. There were no more seizures during her stay in the hospital. As part of the follow-up interview after 6 months, the woman did not report any further episodes and did not smoke the preparation called “Spice” again (15).

In order to inhibit excitation, haloperidol—an antagonist of the dopamine receptors—is also administered. Simmons et al. (17) describe the case of a 21-year-old man who was hospitalized after collapsing on the floor in a store. He presented with repetitive back and forth movements and was unable to speak, but was able to obey verbal commands. The emergency services transported him to the emergency department in an oxygen mask. After being admitted, his blood pressure was 204/103 mmHg, his pulse was 48 beats per minute, respiratory rate 8 breaths/min, and temperature 37.8°C. He had no signs of injury. His skin was warm and dry. Initial laboratory testing showed abnormal leukocyte counts and elevated levels of lactate, glucose, and creatine kinase. A urine drug test and blood alcohol level were negative. Due to the hypoventilation and impaired consciousness, the patient was intubated. Laboratory analysis revealed JWH-018 and JWH-073 synthetic cannabinoid metabolites. The doctors administered 5 mg of haloperidol to sedate him. The next day he was extubated. The leukocytosis, elevated lactate levels, and elevated creatine kinase levels resolved after the administration of intravenous fluids. The man confessed to taking a drug called “Spice,” which likely caused the change in his behavior. He was discharged from the hospital after 24 h (17).

According to the literature on the subject, patients are also given ondansetron (a 5-HT3 antagonist) and promethazine (a histamine receptor antagonist) to control vomiting. Hopkins et al. (20) described a case of a 30-year-old man who presented to the ED with persistent abdominal pain, nausea, and vomiting. His symptoms also did not improve after oral ondansetron, which he had been prescribed at another emergency room just 3 days earlier. He told the story of countless visits to hospitals and clinics over the past few years, mostly at local emergency rooms, some of which resulted in hospital admissions and others in outpatient gastroenterologist consultations. The patient reported that in the previous 2 years he had had “more abdominal CT scans than he could remember,” several abdominal ultrasound scans, and two endoscopic procedures. He also believed that he had severe cramping abdominal pain associated with persistent nausea and vomiting which did not resolve after treatment and recurred every few weeks. His symptoms would last for two or 3 days then slowly subside, often after several visits to the ED for intravenous hydration and antiemetic therapy. In fact, the patient stated that his only real relief was taking hot showers, which meant he bathed several times a day. Further interview revealed that he had been using marijuana since the age of 13 and had smoked several times a day for the past few years, but had stopped using marijuana 6 months earlier. Interestingly, he reported that he had been convicted of growing and possessing marijuana 6 months earlier and was forced to submit weekly urine samples for screening as part of his release. He quickly identified the product with the trade name “Synthetic Sweat” as an ideal substitute because it had similar psychotropic effects but could not be detected by his court-ordered drug testing. Once he was convinced that he could smoke NPSs with impunity, he quickly returned to his daily smoking habits and in the month before the situation in question, he often smoked synthetic cannabinoids every hour, and even woke up several times during the night to smoke. The patient provided a sample of “Synthetic Sweat” which contained a packet of dried herbs that he had purchased from a local grocery store. He reported that he had previously experimented with several brands of synthetic marijuana, including “K2” and “Spice,” which were also available for sale at the same location. However, for the 2 months prior to this episode, he had only been using the “Scooby Snacks” brand. With the use of mass spectrometry and gas chromatography, the herbal mixture was found to contain the following synthetic cannabinoids: JWH-018, JWH-073, JWH-122, AM2201, and AM-694. The patient's urine sample collected at the ED was negative for THC metabolites. It is worth noting that an analysis of his urine by liquid chromatography and mass spectrometry revealed synthetic cannabinoids similar to the herbal mixture which he brought: JWH-018, JWH-073 and AM-2201. His condition improved thanks to IV fluids and intravenous ondansetron. The patient was discharged with a prescription for promethazine suppositories. Three months later, the man was abstaining from marijuana and synthetic cannabinoid products. He reported that it took 2 weeks of sobriety before his symptoms completely subsided. It is noteworthy that the patient reported that after the symptoms had resolved, he no longer required multiple baths and showers every day, his hygiene habits normalized, and his quality of life improved considerably (20).

#### 2014–2015

##### Synthetic Cathinones

Adamowicz et al. (21) describe the diazepam and midazolam treatment of a 20-year-old man with a history of addiction to psychoactive substances. The patient consumed substances that he purchased online, which he combined with 250 ml of 40% alcohol. A few minutes after consumption, he developed severe psychomotor agitation, as a result of which an ambulance was called. The patient was given 10 mg of diazepam and 5 mg of midazolam on site. On admission to the hospital, the man was conscious, cardiovascularly and respiratorily stable, severely agitated, and non-verbal. The patient screamed and fought; he was uncooperative and a threat to his own health. He presented with convulsions and an elevated temperature. His electrocardiogram showed a sinus rhythm. After 2 h, he suffered from cardiac arrest; intubation, ventilation, defibrillation, and cardiac massage were performed and adrenaline and atropine were administered, but this did not have much effect. Death occurred <4 h after the consumption of the psychoactive substance. Postmortem blood tests showed that the young man was intoxicated with 3-MMC, 5-APB, and ethyl alcohol (21).

Midazolam was also used in a case described by Hall et al. (22), in which a 21-year-old man who had snorted “bath salts” (research showed that the product was a mixture of synthetic cathinones) was described by police officers as having “superhuman strength” and being seemingly immune to pain. He was sweating heavily, making growling noises, and hallucinating. Help was called and paramedics immediately administered 10 mg of midazolam intramuscularly. The patient was immobilized and transported to the emergency room. The patient's condition normalized after sedation and intravenous rehydration with saline solution (22). Propofol was cited as an alternative to benzodiazepines in studies done from 2010 to 2013, but more recent research has also demonstrated the effectiveness of dexmedetomidine (23).

Pichini et al. (24) in their article, report the case of a male infant who, 20 h after delivery, began to display increased irritability, high-pitched crying, increased muscle tone of the limbs and increased tendon reflexes. The mother confessed to smoking mephedrone daily during her pregnancy. The newborn was diagnosed with neonatal withdrawal syndrome (NWS). The following treatment was applied: phenobarbital once as an intramuscular bolus at a dosage of 20 mg/kg, and then 10 mg/kg per day orally. The dose was gradually reduced over the following days as the Finnegan score (the neonatal withdrawal symptom score) was above eight. After 12 days of phenobarbital treatment, the symptoms improved. HPLC analysis showed the presence of methadone, its metabolite EDDP (2-ethylidene-1,5-dimethyl-3,3-diphenylpyrrolidine) and 4-MMC (4-methylmethcathinone; mephedrone) in the blood of the newborn. After 15 days, he was discharged from the hospital (24).

Pierluigi et al. (25) describe a case illustrating the use of risperidone in a 28-year-old man in 2014. The patient was admitted to the clinic due to symptoms suggestive of a depressive episode that had occurred within the previous 4 weeks. When he was hospitalized, his assessment of reality was not marked by hallucinations or delusions. The patient complained of a general “lack of energy.” He was medicated with antipsychotics and antidepressants, i.e., risperidone (4 mg/day), venlafaxine (75 mg/day), biperide (4 mg/day), and delorazepam (2 mg/day). During the course of therapy, the patient explained that most of the experimental substance use in his life had taken place over several years, but in the last 8 months prior to admission to the ward, his frequency of taking the psychoactive substance Alpha-PVP (synthetic cathinone) had increased. At the same time, he experienced a worsening of his depression symptoms until the last episode, in which he experienced visual hallucinations, leading to a referral for treatment. The stay in the ward lasted 40 days, during which he participated in a rehabilitation program including individual (every 2 weeks) and group (two or three times a day) psychotherapy sessions as well as psychopharmacological assessment and psychomotor rehabilitation. A significant change in his health status occurred after administration of bupropion (150 mg/day). This drug helped reduce the depressive symptoms caused by the consumption of the psychoactive substance (25). This antidepressant was chosen because of its chemical structure. Bupropion, a substituted cathinone, α-aminocetone, is a dopamine and norepinephrine reuptake inhibitor and an antagonist of the nicotinic acetylcholine receptor. It has also been shown to modulate the levels of certain cytokines associated with inflammatory diseases. Numerous studies have also been conducted to investigate the effectiveness of bupropion in smoking cessation therapy (26).

Oppek et al. (27), however, describe a case of a 29-year-old woman with a history of polysubstance addiction who used significant amounts of bupropion (up to 2.4 g) intravenously to intoxicate herself. Although it is widely believed that bupropion is not addictive, this situation shows us that people prone to addiction can consume it in very large doses daily. Based on this case, Oppek and other authors conclude that administration of bupropion in subjects prone to addiction should be carried out with caution, as its possible impact raises doubts (27).

Literature from 2014 to 2015 contains guidelines—for the first time—on how to counteract serotoninergic syndrome. Apart from intensive cooling of the body and the benzodiazepines mentioned in the studies done from 2010 to 2013, 5-HT2A receptor antagonists should be administered, i.e., cyproheptadine or chlorpromazine (28).

##### Synthetic Cannabinoids

Research carried out from 2014 to 2015 shows that the same treatments were used as in previous years, i.e., benzodiazepines, most often lorazepam, to reduce seizures, agitation, and anxiety (29, 30).

Schep et al. (31) presents a case study of diazepam treatment of synthetic cannabinoids benzodiazepine poisoning. A 23-year-old man smoked a joint containing synthetic cannabinoids (trade name K2) for recreational purposes at home. After 6 h, he was suffering from tonic-clonic seizures with urinary incontinence and damage to his tongue. After 3 h, he took another, smaller dose of the psychoactive substance. Vomiting started: he presented to the hospital with nausea, dry mouth and persistent vomiting. On admission, tests showed no neurological or cardiac aberrations. The patient was discharged, and after 3 h (4 h after taking the substance) he had a second seizure episode. The patient returned to the hospital. He received an intravenous infusion of saline (1,500 ml) and oral diazepam. The man was discharged home. In a later study using the LC-MS (liquid chromatography/mass spectroscopy) method, numerous synthetic cannabinoids were detected in the patient's body, i.e., BB-22, AM2233, PB-22, 5F-PB-22, and JWH-122 (31).

Orsini et al. (32) also mention the use of such benzodiazepines as midazolam in the case of a 41-year-old man who smoked a recreational drug under the trade name “K2” (a mixture of synthetic cannabinoids). After ingestion, the patient had acute hypoxic/hypercapnic respiratory failure caused by acute heart failure. In order to achieve sedation, propofol, fentanyl and midazolam were administered. Intravenous antimicrobial therapy consisting of piperacillin/tazobactam (3.375 g every 6 h) was also used. He was extubated on the 11th day of his stay in the Intensive Care Unit (32).

In order to resolve hallucinations, in previous years patients have been given antipsychotic drugs as mentioned, i.e., haloperidol, olanzapine, and quetiapine (33).

One example is the 27-year-old man described by Green and Kinzie (34). He was admitted to the hospital because he was experiencing hallucinations after smoking a cigarette with synthetic marijuana. The patient received a single dose of 10 g olanzapine to treat psychosis. The therapy was successful and the patient was discharged home (34).

If it is necessary to perform intubation in a patient with synthetic cannabinoids poisoning, and to remember to use non-depolarizing neuromuscular blockers, such as rocuronium (29).

In 2015, the idea appeared of using intravenous lipid emulsion therapy (ILE) in patients who show symptoms of acute synthetic cannabinoids poisoning. It was the only symptomatic treatment of bradycardia in these situations.

Clinical cases of men aged 15, 17, and 19 who took a preparation containing synthetic cannabinoids under the trade name “Bonzai,” in which the ILE therapy was applied (20% lipid in the emulsion) was described by Aksel et al. (35). The first male presented with bradycardia and hypotension. An ILE bolus of 1.25 ml/kg was administered followed by an infusion of 0.50 ml/kg per minute over 60 min. Apart from ILE, no other medications were administered. After the infusion, the bradycardia resolved within 5 min. After a 24-h follow-up period, the patient was discharged home. Another patient presented with ventricular extrasystoles (VES). The patient was treated with intravenous bolus of ILE at a dose of 1.5 ml/kg, followed by an infusion of 0.25 ml/kg per minute over 60 min. On the fifth minute of the ILE infusion, it was observed that the VES rate decreased and the patient's general condition also improved. The patient was discharged after 24-h observation. In the third patient, the EKG showed sinus bradycardia. The ILE infusion therapy was used again, first as a 1.5 ml/kg bolus, then an infusion of 0.25 ml/kg per minute for 60 min. Bradycardia gradually began to resolve and completely resolved after the infusion was finished. The patient was discharged after 24-h observation (35).

##### Synthetic Opioids

In the case of opioid analogs, a similar therapy can be applied as for substances from this group that have been known for years, i.e., substitution treatment. Studies show that such treatment does not markedly affect the prevention or treatment of addiction itself. However, it controls the desire to consume drugs and the amount of side effects caused. The therapy consists in prescribing controlled amounts of longer-acting, but less euphoric, opioids. Currently, methadone is the most commonly used synthetic opioid for this purpose. It is effective in relieving the symptoms of opioid withdrawal and reducing the negative effects of illegal drug use. Individual variability in clinical responses to methadone and dosage requirements depends on several factors, including age, diet, metabolism, protein binding, drugs, genetic variants, and other substances taken (36).

Suboxone has been approved for use in Canada since 2007 and contains a combination of buprenorphine and naloxone in a 4:1 ratio. When used sublingually, only buprenorphine shows partial agonist effect, as naloxone cannot be sufficiently absorbed in this form. When administered parenterally, naloxone causes a withdrawal effect in addicted patients. Therefore, the role of this combination is to ultimately alleviate withdrawal symptoms while at the same time stopping intravenous drug use. The effects of Suboxone are less potent than of full opioid agonists, but it causes less physical dependence than other full agonists. It also has weaker dysphoric effects than methadone, which encourages patients to continue treatment. It also has a threshold effect, meaning that its effectiveness remains constant above a certain dose, thus helping to limit abuse (37).

Helander et al. (38) report cases of intoxication in three young men caused mainly by MT-45 (synthetic opioid). In all cases, the presence of other psychoactive substances was found. Treatment with different doses of naloxone was also applied in all three men. Each of the cases ended in the patient's death (38).

The literature from 2015 also describes the management of an overdose of a synthetic opioid, namely desomorphine, for which supportive care is the main treatment option. Naloxone is useful in treating both acute opioid intoxication and overdose. Symptoms of aggression and psychosis can be treated with sedatives (benzodiazepines, propofol) and antipsychotics [haloperidol or less common drugs such as quetiapine or ziprasidone (39).

Another substance used in substitution therapy is naltrexone, which blocks the euphoric effects of opioids. The initially used oral drug turned out to be ineffective after years, and therefore long-acting injections and subcutaneous implants began to be introduced to the market. However, Naltrexone reduces tolerance to opioids, which increases the risk of overdose if the patient returns to the addiction. According to observations conducted between 2000 and 2003 during the follow-up year, the percentage of deaths associated with the use of naltrexone as a treatment for opioid dependence was up to seven times higher than that of methadone. A fairly new and controversial approach is Heroin-assisted Therapy (HAT). It involves administering injections with a controlled amount of diacetylmorphine, the active ingredient in heroin. However, studies show that HAT therapy turns out to be more effective than methadone treatment in terms of reducing consumption and increasing the intervals between the consumed doses of the substitute (40).

##### The 2C Family of Drugs

Tang et al. (41) describe two clinical cases of intoxication with NBOMe: a synthetic hallucinogen considered to be a derivative of the substituted phenylethylamine of the 2C-I family. The first patient, a 17-year-old man, was admitted to the hospital emergency department with symptoms of confusion, agitation and mild consciousness impairment. Immediately after admission, he developed convulsions and a sympathomimetic syndrome with high blood pressure, tachycardia, sweating, hyperthermia, and dilated pupils. A muscle relaxant was used and intubation was performed. To counteract acute sympathomimetic toxicity, the patient required high doses of intravenous fluids and high titrate doses of intravenous diazepam (17.5 mg in total). The patient's condition was controlled by infusions of midazolam (the maximum rate of 0.28 mg/kg/h) and of rucuronium (0.7 mg/kg/h). Cyproheptadine was given prophylactically as an antidote for possible serotonin syndrome. The patient regained full consciousness 12 h after admission to hospital, was discharged after 5 days, and fully recovered. The second patient was a 31-year-old Chinese citizen with a history of addiction who was admitted to a hospital emergency department with sympathomimetic symptoms: signs of agitation, hypertension, dilated pupils, hyperthermia, intense sweating, and moderate consciousness impairment. The patient developed rhabdomyolysis, decreased liver functions and impaired kidney function. The results returned to baseline after the treatment was applied. The patient was treated with intravenous fluids, intravenous lorazepam as a 2 mg bolus every 5 min, until his sympathomimetic symptoms subsided (12 mg in total). His body was cooled with bags of ice. He was given sodium bicarbonate to alkalize the urine. Despite the doctors' recommendations, he left the hospital at his own request after 3 days of hospitalization (41).

##### Amphetamine-Type Stimulants

Serotonin syndrome can be a side effect of taking amphetamine-type stimulants. Its mild form usually resolves spontaneously or requires only maintenance treatment, while in more severe cases, hospitalization is required. As a rule, agitation and tremors are eliminated with benzodiazepines. In a moderate case of serotonin syndrome, the development of excessively high temperature should be prevented, and in more severe cases, 5-HT2A receptor antagonists, e.g., cyproheptadine and chlopromazine, are used. Sedation and intubation are essential for critically ill patients (42).

Sedation and intubation were used in a patient who took an amphetamine derivative: 2,5-dimethoxy-4-chloroamphetamine (DOC), described by Burish et al. (43). An 18-year-old man presented with hallucinations and agitation which progressed to an epileptic seizure. The patient received 2.5 mg intranasal midazolam which stopped the seizures only briefly. The second administration of the same dosage had no effect. The patient received 2 mg of lorazepam which improved his tonic head movement. The patient was sedated and paralyzed with propofol and rucuronium for intubation. Sedation with propofol at 20 μg/min was continued, and in the next hour the drip was increased to 100 μg/min. As rhythmic toe twitching was observed, he received an additional 9 mg of lorazepam, 20 mg/kg fosphenytoin and 7 mg/h midazolam to the drip. At a later stage of treatment, the drip of midazolam was continued and the drip of propofol was weaned off. Leukocytosis and rhabdomyolysis improved with supportive care, especially with antiepileptic drugs and intravenous fluids. On the second day of his hospital stay, the patient was extubated and developed amnesia. His memory improved on the third day, although his reaction time was still slower (43).

Symptoms of amphetamine psychosis caused by chronic use of this type of substances resolve after the administration of antipsychotic drugs. The new generation olanzapine is of particular importance here due to the increased safety of use. However, these measures do not guarantee that psychosis will not recur (39).

Anderson et al. (44) presents a case of the treatment of schizophrenia relapse induced by the consumption of ethylphenidate and benzocaine. A 30-year-old man took a substance labeled as “el blanco” containing these two substances in liquid form: he mixed the powder with a cola drink. He had a relapse of schizophrenia. The patient's treatment was based on the use of clonazepam 0.5 mg four times a day, as well as haloperidol and lorazepam for acute behavioral and agitation disorders. In antipsychotic treatment, olanzapine was used at a dose of 10 mg twice daily, which was then changed to pipothiazine palmitate 50 mg every 4 weeks. Treatment with clozapine was initiated as his recovery was considered incomplete with previous treatment regimens. The patient responded well to clozapine (44).

##### A Vaccine for Drug Addiction

There are also very unusual methods of treating addictions in the literature that could be used for NPSs available on the market.

The idea was to covalently link addiction-inducing substances to immunoproteins via binding molecules. Scientists believe that the drug molecule itself is too small to trigger the immune response, but such a combination could trigger the production of antibodies that detect the molecule and seek to eliminate it from the body, which would significantly reduce its effects on the central nervous system. Such vaccines against morphine, nicotine, and cocaine have even entered the stage of clinical trials (45).

#### 2016–2019

##### Synthetic Cathinones

According to the literature, it is still very difficult to define a universal method of treating intoxication with cathinones due to the large differences in action and the immense number of NPS's. As there is no specific treatment plan, therapy is symptomatic and supportive. Taking into account the similarity of cathinones to other stimulants, it is recommended to take similar steps in reducing the side effects. When cathinone-induced delirium is suspected, clinicians focus on controlling agitation and complications such as metabolic acidosis. If serotonin syndrome occurs, benzodiazepines and/or cyproheptadine are recommended, just like in the studies published from 2014 to 2015.

As it follows from the publications released between 2010 and 2015, benzodiazepines are still administered orally or intravenously, e.g., diazepam (0.1–0.3 mg/kg body weight), in the case of over-excitation, hyperthermia, aggression, hallucinations or bradycardia and hypertension. Occasionally, higher doses of the drug can be used to achieve and/or maintain a sedative effect. Heart attacks and arrhythmias should be excluded with the use of electrocardiogram (12). Therapy aimed at controlling aggression and agitation still relies on the intranasal or intramuscular administration of midazolam or the intramuscular administration of lorazepam, which can also prevent seizures. As reported in previous years, if there are contraindications to the use of benzodiazepines (e.g., alcohol consumption), administration of propofol or antipsychotics (haloperidol, olanzapine, ziprasidone) should be considered, which may additionally prevent seizures and dysrhythmia. Furthermore, the literature shows that olanzapine is effective in controlling attacks of aggression. Clinicians do not recommend the use of antipsychotics for acute psychosis, owing to the reduction of the seizure threshold and the risk of extrapyramidal symptoms. As medical data show, there are currently no effective pharmacological treatments for synthetic cathinone addiction and withdrawal syndrome. For patients who regularly take synthetic cathinones, symptomatic treatment should be combined with psychotherapy and behavioral therapy (46–48).

##### Synthetic Cannabinoids

The data published in the medical reports show that there is still no specific antidote to intoxication with synthetic cannabinoids. Treatment is symptomatic and supportive. As mentioned earlier, between 2014 and 2015 research on the use of naltrexone in addiction therapy was initiated. It is still continued and, according to the latest literature, there are reports of positive preliminary results of studies with baclofen and naltrexone in the treatment of withdrawal syndrome (49). The chief supportive clinical procedures include intravenous administration of rehydration fluids, potassium, oxygen, and intubation and mechanical ventilation.

In their case series, Hill et al. (50) report a clinical case of a 57-year-old man who fell after consuming four to six cans of high-proof beer and smoking a preparation called “Old spice.” At the time of admission to the hospital, he was severely hypothermic (31°C), with a pulse of 89/min and systolic blood pressure at 50 mmHg. His condition quickly improved after the administration of intravenous fluids. Blood tests showed mild acidosis and increased lactate levels. The presence of MDMB-CHMICA, 5FADB-PINACA, and AB-PINACA was detected in the patient's serum. The patient was discharged from the hospital 8 h after admission, at his request, against the doctors' recommendations (50).

As in previous years, also more recent literature reports indicate that in the case of central nervous system side effects, such as psychosis, hallucinations or agitation, it is recommended to administer benzodiazepines. Research demonstrates that midazolam is the fastest-acting of all benzodiazepines and antipsychotics. Clonazepam is recommended for the treatment of permanent visual impairment caused by synthetic cannabinoids. Coppola et al. (51) report the case of an 18-year-old man whose hallucinations and visual disturbances completely resolved only a few years after consuming a synthetic cannabinoid. The patient ingested the JWH-122 drug. Symptoms of a synthetic drug poisoning were similar to natural cannabinoids poisoning, with the exception of tachycardia and visual disorders. Acute intoxication symptoms disappeared within 3 h. Over the following days, the patient experienced hallucinations again, saw colorful, geometric forms, and presented with dissociative symptoms. The man was given 6 mg of clonazepam daily, which reduced the frequency and severity of his symptoms. For the next 3 years, the patient occasionally experienced some side effects of acute intoxication, which completely resolved 4 years after the consumption of JWH-122 (51).

With regard to antipsychotics, it should be remembered that, according to the literature, they can prolong the QT interval and interfere with the body's ability to cool itself. Clinical studies indicate that the occasional use of an antipsychotic such as haloperidol controls agitation and psychosis when high doses of benzodiazepines are administered. In addition, the combination of haloperidol and benzodiazepines helps to effectively soothe the patient (52, 53).

Bonaccorso et al. (54) describe the case of a 32-year-old woman with diagnosed schizoaffective disorder, whose condition was stabilized after administering 30 mg of aripiprazole daily and 800 mg of lithium carbonate nightly. The urine toxicology test was negative. After 4 weeks, the patient's condition deteriorated significantly: she displayed a delusional mood and complex delusions of grandeur and persecution. She became physically and verbally aggressive and developed sexual disinhibition, and a repeat urine toxicity test was positive for THC and synthetic cannabinoids. An intensive pharmacological treatment plan was implemented with the administration of 10 mg of haloperidol and 8 mg of clonazepam daily. The patient remained in poor condition for 72 h. After 10 days, the urine drug test was consistently positive for synthetic cannabinoids, confirming the suspicion that the patient had been using drugs in the ward. After the suspension of her permit and more stringent searches, the woman's condition improved significantly. Another case concerned a 20-year-old man who was diagnosed with the first psychotic episode after taking a multi-component substance. The patient was given 50 mg haloperidol decanoate and 10 mg haloperidol per night. He developed over-excitation, sexual disinhibition, over-aggression, and severe thinking disorders and the urine drug test was positive for synthetic cannabinoids. The patient was therefore treated with 9.75 mg of aripiprazole three times a day and 6 mg clonazepam daily in divided doses. Within 72 h, his condition improved (54).

Patients with symptoms of poisoning are also often given antiemetics such as diphenhydramine or Ondansetron. The use of antidepressants has also been observed. If neuromuscular transmission disorders occur, antiepileptic and anticonvulsant drugs are administered, i.e., Levetiracetam, phenytoin, and valproic acid. Sometimes hypnotics (hydroxyzine, etomidate, zopicon) and anesthetics (vecuronium, lidocaine) as well as pressure-lowering drugs (nitroglycerin, metoprolol, and clonidine) or sympathomimetics such as adrenaline, noradrenaline or phenylephrine are used (55).

In their case study, Hill et al. present the case of a 23-year-old man with a history of recreational drug consumption, including synthetic cannabinoids, marijuana and diazepam. The patient was additionally taking prescription quetiapine. At the time of admission, he exhibited abnormal behavior: hallucinations, instability, sweating, agitation, aggression, insomnia, and a tendency for self-harm (hitting the head against a wall). He was very aggressive in the hospital and refused to cooperate. He had fever, tachycardia, dilated pupils and mild metabolic acidosis. He received 3 mg of lorazepam intravenously. Afterwards, he was intubated and mechanically ventilated. The urine test was positive for benzodiazepines only. In order to maintain normal blood pressure, the patient was given phenylephrine followed by noradrenaline. The day after admission, he was extubated and disconnected from mechanical ventilation. The patient remained confused and aggressive for several consecutive days; he required high doses of benzodiazepines and haloperidol. He was discharged from the hospital after 9 days elapsed from admission. He confirmed the ingestion of a substance called “Vertex.” MDMB-CHMICA and methiopropamine were found in his serum (50).

The Psychoactive Surveillance Consortium and Analysis Network is a network of academic emergency medical services, toxicologists, and pharmacologists that collects clinical data paired with biological samples to identify and improve treatment methods for NPS poisoning. Brandehoff et al. (56) provide data on eight reported use cases of “Black Mamba.” The authors describe cases of intoxication in four women and four men with a median age of 28, who experienced acute symptoms such as agitation, delirium, chest pain, and heart rhythm disturbances visible in the electrocardiogram. One patient with a prior seizure also experienced a tonic-clonic seizure. Almost all patients had high blood pressure, only two of them developed tachycardia and two patients also developed hypokalemia. The patients received diazepam or lorazepam for sedation. One patient was also given haloperidol and diphenhydramine in addition to lorazepam. In four of the subjects, the agitation subsided. Some patients also received ondansetron for nausea. A 25-year-old woman in whose serum no other drugs such as cocaine or methamphetamine were found, developed severe agitation and aggression. Her blood pressure was 140,106 mmHg and she also developed tachycardia. The woman was administered 2 mg of lorazepam, 5 mg of haloperidol and 50 mg of diphenhydramine. The patient was discharged after 10 h of observation in the emergency department. Another case was a 23-year-old woman who complained of chest pain. An electrocardiogram showed T wave inversion, nausea and diarrhea. Her blood pressure was 148/100 mmHg. The patient received 15 mg of ketorolac, 4 mg of ondansentron and 1 g of acetaminophen, as well as 30 ml of simethicone and 15 ml of 2% viscous lidocaine. Having been observed for 9 h in the emergency department, she was discharged home. A 27-year-old man who was taking marijuana concurrently with a synthetic cannabinoid complained of bilateral palpitations, numbness in his hands and vomiting; his blood pressure was 155/85 mmHg. The patient was administered 1 mg of lorazepam twice, as well as 1l mg of saline solution. He was discharged from the hospital after 3 h (56).

The literature also shows that regardless of whether the patient has experienced psychosis before, the use of atypical antipsychotics, such as ziprasidone, olanzapine or zuclopenthixol, may be effective. Bonaccorso et al. (54) describe the use of zuclopenthixol in the pharmacotherapy of a 39-year-old man with a long history of bipolar disorder and abuse of many psychoactive substances (alcohol, cocaine, MDMA, marijuana and legal highs). During his admission at Highgate Mental Health Center, the patient was highly agitated and aggressive. The urine drug toxicity was negative. He was administered 400 mg of Ablify Depot, but the drug showed little therapeutic efficacy. The man was diagnosed with a manic episode of bipolar disorder. Therapy with Ablify was discontinued, zuclopenthixol was started and the medications previously taken by the patient were continued. During the patient's 3-week stay at the center, urine drug testing was positive for synthetic cannabinoids, benzodiazepines and THC, and the patient showed poor compliance with treatment. After his permit was suspended, the urine drug test was negative 1 week later and the episodes of aggressive behavior resolved. Therapy with 300 mg of zuclopenthixol weekly and 1,200 mg of sodium valproate was satisfactory (54).

The side effects of the medications administered may be more pronounced in patients who already suffer from psychosis, agitation or anxiety. The emerging akathisia, which is one of the side effects of these drugs, may increase the already existing anxiety and may be confused with the stimulation resulting from the use of synthetic cannabinoids, which may lead to incorrect pharmacotherapy. The literature on the subject draws attention to the fact that first-generation antipsychotics administered with or without anticholinergics, or with benzodiazepines, are superior to benzodiazepine monotherapy in patients with acute psychotic symptoms such as delusions, hallucinations and agitation (57). Sweet et al. (58) report a case of a 47-year-old man who ingested a synthetic cannabinoid “King Kong,” and then was brought by the police to the emergency department with high fever, tachycardia, accelerated breathing and blood pressure of 153/103 mm Hg. The man was agitated, had delusions and hallucinations. He was given 1 olanzapine tablet orally, but it was not possible to calm him down, he was still aggressive toward the medical staff. For this reason, the patient was given 10 mg of haloperidol intramuscularly together with 2 mg of lorazepam and 50 mg of diphenhydramine. The next day he was calm and able to take part in a medical interview. He admitted that he had smoked King Kong before and was involuntarily hospitalized each time (58).

Bonaccorso et al. (54) report the case of a 28-year-old man with a 4-year-long psychosis with frequent relapses, who was treated with risperidone at a dosage of 37.5 mg every 2 weeks + olanzapine 10 mg daily + pregabalin 100 mg daily. After the patient was transferred to the intensive care unit (PICU), previous treatment was continued. The patient's condition was stable and the drug test was negative. One week after the patient's transfer, his condition deteriorated: he developed persecutory delusions and became verbally and physically aggressive. After the patient was transferred, a repeated toxicology test was positive for synthetic cannabinoids. The olanzapine dosage was increased to 20 mg and clonazepam to 8 mg, which improved the patient's condition within 24 h (54).

As for the circulatory system, in the case of chest pain, specialists recommend excluding myocardial ischemia and arrhythmias using an electrocardiogram. As indicated in the literature, bradycardia may require external cardiac stimulation or administration of atropine. Medical data indicate that tachycardia should be treated with benzodiazepines and intravenous fluids (59). For cardiac ischemia, administration of antiplatelet drugs such as clopidogrel, aspirin or nitroglycerin is recommended (60).

There is clinical evidence that an overdose of synthetic cannabinoids can directly or indirectly lead to death. A systematic review from 2016 found that out of 4,000 cases, at least 26 were fatal, and death was associated with the consumption of synthetic cannabinoids (12). Due to the short duration of the effects of these substances, hospitalization usually lasts several hours (42, 50).

In reports from 2017, we can find an example of a multicenter cohort study focused on the analysis of patients who take synthetic cannabinoids. The study was based on cases from the ToxIC Case Registry. This register is kept by specialists in their field and therefore it should be assumed that it constitutes a reliable database. The study in question covers 353 cases of synthetic cannabinoids poisoning. The median age among patients was 25, most of whom— 84%—were men. As many as 61% of the patients were referred to the emergency room, 15% were admitted to the hospital room, and 24% to the ICU (61).

According to the study, the most common symptoms of SC poisoning in patients are:

- agitation, delirium and psychosis in 41% of patients
- coma or depression of the central nervous system in 24.1% of patients
- epileptic seizures in 17% of patients.
- tachycardia as 12.5% of patients had a heart rate above 140 beats per minute
- hallucinations in 7.1% of patients
- bradycardia as 5.7% of patients had a heart rate below 50 beats per minute.

Moreover, symptoms such as hypotension, hyperthermia, rhabdomyolysis and kidney damage occurred. As far as pharmacological treatment is concerned, the most frequently used drug therapy was benzodiazepines (37%), followed by antipsychotics (10%). About 9% of patients were medicated with both benzodiazepines and antipsychotics. Naloxone was administered in 3.4% of cases, while 2.5% of subjects received anticonvulsant treatment and 2.3% received neuromuscular blockers and mechanical ventilation. Only three out of 15 severely hypotensive patients required vasopressors. One patient died (61).

There is also data on the possibility of treating cannabis dependence with the use of preparations containing tetrahydrocannabinol, gabapentin or N-acetylcysteine, but there are no studies confirming their effectiveness in the treatment of addiction to synthetic cannabinoids (57).

##### Synthetic Opioids

It is generally recognized that the real antidote only exists for cases of opioid poisoning. The literature clearly indicates that in opioid poisoning, the most important course of action is to inhibit respiratory depression with the μ-naloxone opioid receptor antagonist (62).

Due to the ease of overdose and the very fast action of the opioid, data also suggest that appropriate treatment procedures should be implemented as soon as possible. If an overdose is suspected, treatment should be initiated when the respiratory rate is <12 per minute and the oxygen saturation is <90%. Initially, oxygen should be given together with mechanical ventilation (if necessary): these procedures are designed to open the airway. The next step is the administration of an opioid antagonist to reverse respiratory failure (36). Naloxone can be administered by any route: intravenous, intramuscular, intranasal, subcutaneous, intratracheal, inhalation, and sublingual. Orally administered naloxone undergoes first-pass metabolism, which is associated with its low bioavailability, therefore the parenteral route is chosen much more often. Naloxone administered intravenously works as early as after 30 s. Intranasal administration is also gaining more and more favor, due to the ease of application and the reduced risk of needle stick injuries to medical personnel. There is no specific dosage recommended for respiratory depression. The dosage used depends on the type of opioid overdose, the patient's weight and the substance amount taken, and it may be necessary to administer naloxone several times at intervals (53). According to the literature, the initial dose for children is 0.1 mg/kg (35). In the case of out-of-hospital administration, it is assumed that 400 μg of naloxone should be intravenously injected initially and constantly continued at a dose of 400 μg every 2–3 min, until vital functions are restored or the dosage should be stepped up to 2 × 0.8 mg. The maximum allowable dose of naloxone in this situation is 2 mg. When administered in the hospital, intravenous starting dose is 400 μg, if there is no response within 60 s, then 800 μg. If there is still no response, another 800 μg dose should be given after 60 s. If there is still no effect, a final dose of 2 mg is administered (in exceptional cases, in the absence of a satisfactory effect, a dose of 4 mg is used (42).

Wilde et al. report a case of a 25-year-old male after intranasal intake of cyclopropylfentanyl in 2019. The man snorted a preparation that was supposed to contain fentanyl. Ten minutes later, he developed nausea, sweating and shortness of breath, followed by coma and respiratory failure. On arrival, the rescuers gave the patient oxygen and 0.4 mg of naloxone. Another dose of 0.4 mg of naloxone was required to restore respiratory function. Upon admission to the hospital, the patient was in a coma, with severely constricted pupils and a respiratory rate of 14/min. and additionally, hypothermia (34.9°C) was noted. The patient was observed in the intensive care unit and oxygen was administered intranasally. Within 12 consecutive hours, he experienced episodes of apnea with oxygen desaturation. After 1 day under observation, he was discharged from the hospital. Immunoassays showed positive results for cocaine, cannabinoids, LSD and cyclopropylfentanyl consumption (63).

According to the data from the Chicago Emergency Department, during the deadly wave of fentanyl abuse between 2005 and 2006, the standard dose of 0.4 mg naloxone was effective in only 15% of cases, and the mean dose of naloxone required to save lives was 3.36 mg. Initial therapy should be targeted at restoring respiratory function, but should not be focused on the patient regaining consciousness. The length of hospitalization in an opioid overdose is still under discussion. It is assumed that the patient should be observed for at least 2 h after respiratory function is restored and consciousness is regained since the duration of action of naloxone is between 30 and 120 min. After this time, re-administration of the drug may be required if large amounts of the narcotic substance have been ingested (64).

In the case of MT-45 poisoning, traditional doses of naloxone are also used. In a group of five people who were intoxicated with U-47700 and MT-45, two subjects did not require naloxone, one person improved after administering a dose of 0.4 mg, two cases required 2 mg, and in one person had to have two doses of 2 mg administered (52). However, administration of naloxone in the event of intoxication with synthetic opioids is not always recommended.

Domanski et al. (65) describe a clinical case of a 26-year-old man and a 24-year-old woman who, according to clinical findings, consumed alcohol and alprazolam in combination with a powder that was supposed to be synthetic cocaine, but turned out to be a new U-47700 opioid. Three hours after intake, the man was found unconscious with severe breathing problems, cyanosis and oxygen saturation at the level of 50%. At first he was intubated and placed in an orthopedic collar. He was given ketamine, lorazepam and rocuronium during intubation. The doctors decided not to administer naloxone because according to available information he had ingested alcohol concomitantly with alprazolam. The patient had pinpoint pupils, heart rate of 125 beats/min, blood pressure of 150/63 mmHg, and 14 bpm. Initially, oxygen saturation was 84%, but as a result of artificial ventilation, it quickly increased to 100%. Laboratory tests showed mild acute kidney injury (creatinine 1.5 mmol/L) and elevated lactate 4.4 mmol/L, which were stabilized after intravenous fluid administration. The ECG revealed a sinus tachycardia of 125 bpm with normal intervals and non-specific ST segment changes. The patient was referred to the intensive care unit, where he was given propofol. He was discharged from the hospital on his request after 3 days of hospitalization (65).

As in previous years, the literature data on patients who overdosed on the U-47700 synthetic opioid indicate the effectiveness of 5 mg diazepam, potassium and leveticeram administered in patients with a depressive mental state. In the case of an overly agitated patient with chest pain, it is advisable to administer saline, 25 mg of diazepam, 15 mg of ketorlac, lisinopril, and 5/325 mg of hydrocodone. Two mg of lorazepam, 5 mg of haloperidol, and 50 mg of diphenhydramine were given to a patient showing agitation and aggressive behavior (66).

According to the case reports, constipation in such patients can be treated with pantoprazole. For withdrawal symptoms, diazepam, pyritamide, clonidine, clomethiazole, and pipamperon are used (67).

The most common therapy is treatment with opioid agonists (AOT). As it was done from 2014 to 2015, methadone and buprenforine are the most commonly used drugs (36, 53).

##### The 2C Family of Drugs

There is no doubt in the literature that, as with other NPS, there is no specific antidote against NBOMEs. As in the reports from 2014 to 2015, in order sedate the patient, reduce aggression, tremors and convulsions, clinicians recommend benzodiazepines, especially lorazepam or midazolam administered intravenously. Sometimes catecholamines (e.g., noradrenaline, dopamine) are prescribed to control bradycardia. As indicated in the published studies, antiarrhythmic drugs (B-blockers, amiodarone) should be administered in the case of supraventricular tachycardia, paired with antipyretic drugs or mechanical cooling if hyperthermia occurs. Blood transfusions may be required in patients with hematological disorders. Furthermore, in the event of rhabdomyolysis, which can lead to serious complications, the literature indicates that skeletal muscles relaxants (e.g., midazolam, rocuronium) are used and fluids are administered at the same time to maintain the urine flow at 200–300 ml/h. Moreover, urine should be alkalized to prevent hyperkalemia and hypocalcaemia and the deposition of myoglobin in the renal ducts (68).

Zygowiec et al. (69) report a case of a 27-year-old man with symptoms of 25C-NBOMe poisoning. The man was brought to the emergency department by the police due to his aggressive behavior. The patient's initial blood pressure was 139/90 mmHg, his heart rate was 146 bpm, and his respiration rate was 28 bpm. He was given 2 liters of 0.9% saline via an intravenous solution. The case was consulted with specialists from the Michigan Poison Control Center. They recommended intravenous fluids, cardiopulmonary monitoring, and administration of benzodiazepines as needed. The patient was initially administered 2 mg of lorazepam and 5 mg of haloperidol lactate. The next day, the man escaped from the hospital, but after about 3 weeks, he returned to the emergency department with symptoms of intoxication with 25I-NBOMe. Upon admission to the ward, the patient was very agitated, his heart rate was 120 beats per minute, his blood pressure was 153/119 mm Hg, and his respiratory rate was 22 breaths/min. The patient was intravenously treated with 4 mg of lorazepam and 1 L of 0.9% saline. The dosage of benzodiazepines was gradually increased. Within 4.5 h, he received a total of 34 mg of lorazepam. Despite high doses of lorazepam, the patient's vital signs remained stable and he did not require airway support. He was admitted to hospital for further psychiatric monitoring and evaluation. The psychiatrist recommended that he be transferred to an addiction center. The patient, however, refused and requested to be discharged from hospital 48 h after admission (69).

### PCP—Phencyclidine Derivatives

According to the literature, due to the lack of specific treatment methods, the treatment of PCP poisoning is symptomatic. Some of the patients require intensive care. As the reports show, the main treatment regimens include administration of benzodiazepines (midazolam) and of intravenous fluid (NaCl solution). Berar et al. (70) describe a case of a 17-year-old male who ingested 3-McO-PCP. The man with a history of drug consumption was admitted to the hospital with severe agitation and altered states of consciousness. Afterwards, the patient experienced nystagmus with dysarthria. His systolic/diastolic pressure was 158/131 mmHg, his HR was 100 beats/min, and his oxygen saturation was 99%. Laboratory tests showed hyperlactatemia (2.6 mmol/L), elevated creatine kinase levels (CK 290IU/L) and respiratory acidosis. Due to hypertonia, the patient was given 1 mg of midazolam and 1500 ml of saline solution. He was referred to the intensive care unit, where, in order to control anxiety and agitation, he received antipsychotic drugs: loxapine and cyamemazine. Additionally, the intravenous administration of electrolytes 3,000 ml/24h was continued. Within 24 h, neurological disorders, blood pressure and heart rate returned to baseline. The following day, the patient only experienced the effects of moderate sedation. The interview showed that the man consumed about 200 mg of a substance called 3-MeO-PCP. He was referred to a psychiatric hospital, from which he was discharged after a few days. One week later he was hospitalized again after ingesting 50 mg of the same substance. On admission to the hospital, his vital signs were as follows: blood pressure 150/104 mmHg, HR 105 bpm, SpO2 96%. Mild psychomotor disorders and dysarthria were observed. The patient was discharged from the hospital after 12-h observation (70).

Zidkova et al. (71) report two cases of 3-methoxyphencyclidine poisoning. A 37-year-old man was admitted to the emergency department 2 h after consuming the drug with alcohol. The patient presented with symptoms such as high blood pressure (170/100 mm) and tachycardia (120/min). He also developed psychosis, altered neurological function, and increased muscle tone. The patient underwent gastric lavage, was given activated charcoal, macrogol and prokinetic drugs: metoclopramide and synostigmine. The patient developed hypophosphatemia (0.55 mmol/L), but the kidney and liver function was normal. Within hours, he regained full consciousness, and showed no neurological or hemodynamic symptoms. He reported complete amnesia from the period of intoxication. Having been observed for 24 h in the emergency department, the patient was discharged home (71).

Dunlop et al. (72) describe a clinical case of a 56-year-old male who ingested 3-OH-PCP and hexene. A patient with a history of a heart attack and one stent in a coronary artery was regularly taking clopidogrel, simvastatin, atenolol, and lansoprazole. He was found at home. When the paramedics arrived, he was drowsy, sweaty, and hot, his pupils were rigid and insensitive. His vital signs were as follows: RR 40 breaths/min, body temperature 39.9°C. In an interview, he admitted that he had ingested a substance called hexene. He was hallucinating and sweating. Within 4 h, the patient was administered intravenously 4.3 l of infusion fluids in crystalline form, which had previously been cooled to 2–6°C. Additionally, the patient received 7.5 mg of diazepam intravenously within 2 h. His body temperature dropped to 37.7°C after 1 h of treatment. On the second day of hospitalization, the patient's clinical condition improved. His history showed that he had been using N-ethylhexedrone by nasal insufflation and rectal injection for 3 months. He admitted that he developed a psychological addiction to it. The night before admission to the hospital, he took 100 mg of hexene, but did not experience any negative effects. In the morning he felt as if he had an upper respiratory tract infection, so he took a cough syrup of unknown composition and about 10 mg of 3-OH-PCP. He did not remember what happened until the second day of his stay in the hospital. The patient developed rhabdomyolysis with moderately severe kidney damage. He was discharged from the hospital after 25 h (72).

Additionally, it has been proven that in the case of phencyclidine derivatives poisoning, flumazenil and naloxone do not show effective action. There are reports in the literature about the beneficial effects of Noopept, but they have not yet been scientifically proven. Hemodialysis and peritoneal dialysis seem to be ineffective in PCP poisoning (70, 72).

### Designer Benzodiazepines

Designer benzodiazepines are a group of substances whose pharmacotherapy case reports have only recently appeared in the literature. The most common therapeutic steps include intravenous fluid administration, naloxone, oxygen, other benzodiazepines, and flumazenil. Additionally, intubation, mechanical ventilation and administration of antiemetics are sometimes used. In most cases, there is a reduction in side effects and treatment is found to be effective. Many patients end up in intensive care units, and some of them are referred to psychiatric centers. Deaths are very rare. The mean hospitalization time and the mean duration of drug-related adverse events are <24 h. In extreme cases, the effects may last up to 1 month. Flumazenil is a commonly reported benzodiazepine antidote in studies. It is an antagonist of benzodiazepine receptors. It shows weak internal action on GABA-a receptors. According to the literature, it is used to reverse sedation and to prevent respiratory depression caused by benzodiazepine consumption (73, 74).

Gummin et al. (75) report a fatal clinical case of a 24-year-old man who ingested flubromazolam. The man was found unconscious with a half empty drug wrapper. He was intubated en route to the hospital. The medical interview showed schizophrenia and abuse of alcohol and other substances. Additionally, the patient was taking medications such as risperidone, alprazolam, zolpidem, popranolol, atomoxetine, disulfiram, lithium, and clozapine. Benzodiazepine immunoassays were positive. On day six, the patient developed fever (38.6°C) and was treated with meropenem and fluconazole. On day 12, he was transferred to a specialist hospital. On day 15, tracheostomy and percutaneous endoscopic gastrostomy were performed. Additionally, leviracetam was included in the treatment. The patient received scopolamine combined with albuterol and ipratropium by nebulization. On day 19 the patient was insensitive to pain stimuli and his pupils were dilated and non-reactive. On day 20, as a result of non-response to treatment and deterioration of his condition, flumazenil was administered, but without the expected improvement. On the 21st day, he was transported to a hospice, where on the 30th day he died. Autopsy revealed that the cause of death was a complication of drug overdose (75).

### Statistical Analysis

The statistical analysis was carried out using the IBM SPSS Statistics 25 package. The chi-square test was used to analyze whether the compared groups of people were equinumerous and to check whether there was a statistically significant relationship between the nominal variables. Logistic regression was applied to determine whether consumption of the most frequently used NPS was a statistically significant predictor of the occurrence of characteristic clinical symptoms and death. Spearman's correlation was used to analyze whether there was a statistically significant relationship between the publication year for the case reports and the total number of NPS used.

## Results

### Clinical Characteristics

The study included 51 NPS case reports written between 2010 and 2019 (Table 1).

**Table 1:** Case reports on the use of various types of new psychoactive substances (NPS) published between 2010 and 2019.

**References**	**New psychoactive substance**	**Sex**	**Age**	**The effect associated with the intake of NPS**	**Treatment method**	**The effect of pharmacotherapy**
(14)	Mephedrone	Male	22	Tachycardia, diaphoresis, hypertonia, hyperreflexia, clonus	Intravenous fluids, diazepam	Fifteen hours after admission, he was discharged from hospital after his neurological symptoms subsided.
(76)	“Spice”	7 men	13-27	Confusion, convulsions, daze, nervousness	Benzodiazepines	–
(8)	“Bath salt” Mephedrone	Male	–	Hyperactivity, insomnia, anger, anxiety, hallucinations	Risperidone 0.5 mg	After 2 days of therapy, the symptoms of paranoia subsided and the patient was discharged home after 5 days.
(8)	“Bath salt” Mephedrone	Female	–	Anorexia, anxiety, insomnia, hallucinations	Risperidone 0.5 mg	After 3 days of therapy, anxiety and behavioural withdrawal subsided. After 2 months, the patient complained about memory loss when taking mephedrone.
(8)	“Bath salt” Mephedrone	Male	–	Hyperactivity, insomnia, visual and auditory hallucinations	Haloperidol 1 mg	After 3 days, the patient no longer suffered from hallucinations and hyperactivity. The patient's friend pointed to his memory distortion when taking mephedrone.
(6)	MDPV “Powdered rush”	Female	27	Tachycardia, diaphoresis, anxiety, psychosis	Risperidone 0.5 mg	The patient's condition stabilised the next day after taking the medicine.
(17)	“Spice” (JWH-018)	Male	25	Dilated pupils, uncontrolled movements, no reaction to external stimuli, tachycardia	Lorazepam 4 mg	Three hours after taking the medicine, tachycardia subsided and the patient's condition improved.
(17)	JWH-018, JWH-073 “Spice”	Male	21	Confusion, uncontrolled movements of the lower extremities, high blood pressure, low heart rate, respiratory disorders; the patient was intubated	Haloperidol 5 mg	The next morning, the patient was extubated, 24 h after admission he was discharged from the hospital.
(18)	“Funky monkey Spice”	Male	24	Erectile dysfunction, tonic-clonic seizure with urinary incontinence; abnormalities were found during magnetic resonance imagining (MRI)	Hydrocodone	Thirteen days after taking the medicine, the MRI examination was performed again, all abnormalities resolved.
(18)	“Black mamba Spice”	Female	36	Epileptic seizure, intubated	Lorazepam, etomidate, vekuronium, propofol, levetiracetam, phenytoin	Twelve hours after taking the medicines, the patient was extubated, no further seizures.
(77)	“Spice” THC, JWH-018, JWH-073	Female	22	Confusion, anxiety, tremor, palpitations	–	The patient was calmed down and observed for an hour, then released home.
(77)	“Spice” THC, JWH-018, JWH-073	Female	20	Confusion, anxiety, increased pulse	–	The patient refused to undergo laboratory tests. She left the hospital in good condition.
(15)	“Bath salt” Mephedrone	Male	32	Tachycardia, hallucinations, premature ventricular contractions	Lorazepam 6 mg haloperidol 5 mg	The patient gradually became calmer, but he still had impaired consciousness and orientation. Thirty-five hours after taking EMS, the patient was still irritable, but his mental condition improved significantly. Over the next 24 h, the patient's condition improved, he was discharged from the hospital after all symptoms disappeared.
(15)	“Bath salt” Mephedrone	Male	26	Auditory hallucinations, a sense of detachment from reality, paranoia, suicidal thoughts	Lorazepam 5 mg risperidol 2 × 0.5 mg	Ninety-six hours after taking the medicines, he was discharged from the hospital. The patient was conscious, hallucinations subsided.
(76)	K2	Male	17	Aggression, convulsions, drowsiness, daze	Naloxone 2 mg	–
(13)	Cathinone	Male	23	Depression	Bupropion, a dose gradually increased to 450 mg daily	After 4 weeks of using bupropion at a dose of 300 mg/day, the patient reported a partial improvement in mood and a decrease in thirst for cathinones. He used bupropionin at a dose of 450 mg/day for the next 4 weeks, after which the patient reported complete abstinence and a significant improvement in mood.
(12)	“Bath salt”	Female	26	Suspiciousness, hallucinations, social withdrawal	Haloperidol and risperidol (no improvement) 120 mg of lurasidone overnight (minimal improvement) 40 mg of citalopram 300 mg of trazodon overnight Electroconvulsive treatment was also used.	After electroconvulsive treatment, the number of hallucinations decreased, mood improved and social anxiety decreased. After 8 months of treatment, the patient occasionally had hallucinations, but she no longer suffered from mood disorders.
(10)	Mephedrone, MDPV, benzocaine, caffeine, lidocaine, procaine (“Cristallus”)	Male	22	Tachycardia, daze, aggression, significant increase in troponin, creatine kinase and creatinine levels	Lorazepam 2 mg	The levels of troponin and creatinine decreased, the level of creatinine kinase increased, but the patient did not complain of muscle pain.
(16)	“Bath salts”	Male	36	Paranoia, anxiety, hallucinations, chaotic speech, aggression, dilated pupils, flushed skin, diaphoresis, high pressure, uncontrolled movements (waving, jerking), fast heart rate	Midazolam, lorazepam, etomidate, rocuronium, sodium thiosulfate	No improvement after midazolam and lorazepam. The patient was intubated and received etomidate and rocuronium. Poisoning with cyanide or poisonous alcohol was suspected, and sodium thiosulfate and fomepizole were administered for this purpose.
(20)	JWH-018, JWH-073, AM-2201	Male	30	Abdominal pain, nausea, vomiting	Ondansetron, promethazine	After receiving fluids and ondasetron, the patient's condition improved. He was discharged from the hospital, but only after 2 weeks of sobriety did all symptoms disappear.
(9)	“Bath salts”	Male	15	Stimulation, psychotic symptoms, paranoia (the patient barricaded himself at home), periods of psychomotor impairment	Olanzapine 5 mg daily, then increased to 7.5 mg lorazepam 0.5 mg twice daily	Symptoms of paranoia subsided after 3 days. After returning home, the patient took 7.5 mg of olanzapine daily and 0.5 mg of lorazepam twice daily.
(24)	Mephedrone EDDP	Male	Newborn baby	Nervousness and irritability, high scream, increased muscular tension in the extremities, increased tendon reflexes	Phenobarbital 20 mg/kg once intramuscularly, then 10 mg/kg daily orally	After 12 days of phenobarbital treatment, the symptoms improved. The infant was discharged after 15 days weighing 2,566 g and being in good condition.
(41)	25B-NBOMe 25C-NBOMe	Male	17	High blood pressure, tachycardia, diaphoresis, hyperthermia, dilated pupils	Diazepam (by titration, total 17.5 mg) midazolam (infusion 0.28 mg/kg/h) rocuronium (infusion 0.7 mg/kg/h) cyproheptadine	The patient regained consciousness 12 h after admission to hospital and was discharged after 5 days, fully recovered.
(41)	25B-NBOMe 25C-NBOMe	Male	31	High blood pressure, tachycardia, diaphoresis, hyperthermia, dilated pupils, rhabdomyolysis	Lorazepam intravenously 2 mg every 5 min (total 12 mg) sodium bicarbonate	Despite the doctors' recommendations, the patient left the hospital after 3 days of hospitalisation on request.
(21)	3-MMC (3-methylcathinone) 5-APB Ethanol	Male	20	Psychomotor agitation, accelerated heart rate, convulsions, increased temperature, cardiac arrest	Diazepam (10 mg) midazolam (5 mg) adrenalin atropine intubation, ventilation, defibrillation, fatal case	The patient died <4 h after taking the substance.
(31)	BB-22 AM2233 PB-22 5F-PB-22 JWH-122	Male	23	Seizure with urinary incontinence, nausea, dry mouth	Saline (1,500 ml intravenously) Diazepam (orally)	The seizures stopped. The patient was discharged from the hospital.
(22)	MDPV	Male	21	Insomnia, increased physical strength, no pain, delusions, aggression, hallucinations	Midazolam (10 mg intramuscularly) saline hydration	Blood pressure returned to normal after 12 h. Renal function and blood parameters stabilised after treatment.
(38)	MT-45 Methiopropamine PCP 3-MeO-PCP	Male	26	Loss of consciousness, apnea, bruising, dilated pupils, hearing impairment	Naloxone 0.4 mg fatal case	The patient died.
(38)	MT-45 Benzofurans Flubromazepam Pirazolam a-PVP	Male	32	Decreased consciousness, low saturation, sight impairment	Naloxone 0.1 mg fatal case	The patient died.
(38)	MT-45 3-MMC Pyrazolam	Male	24	Decreased consciousness, low saturation, sight impairment	Naloxone 3 × 0.4 mg (total 1.2 mg) fatal case	The patient died.
(32)	K2	Male	41	Acute hypoxia, hypercapnia, respiratory failure resulting from acute congestive heart failure, ST-segment elevation myocardial infarction	Propofol, fentanyl and midazolam to achieve adequate sedation and synchronisation of ventilation, antimicrobial therapy consisting of piperacillin and tazobactam for aspirative pneumonia	The patient was successfully extubated on the 11th day after admission to the intensive care unit (ICU).
(43)	DOC	Male	18	Seizures, left eye deflection, dilated pupils and abnormal movement of the limbs, tonic turning of the head and eyesight	Midazolam 2.5 mg intranasally to stop the attack temporarily, lorazepam 2 mg intravenously to improve tonic head movement, propofol for sedation, starting from 20 μg/min and increasing to 100 μg/min in the next hour additionally: lorazepam 9 mg, phosphenytoin 20 mg/kg intravenously and midazolam at a rate of 7 mg/h for drip	The patient was successfully extubated on the second day in hospital, had amnesia, neurological tests were normal; his memory improved on the third day, but he still had a slower response time.
(35)	Synthetic cannabinoid (“Bonzai”)	Male	19	Sinus bradycardia	Bolus 1.5 ml/kg, 20% of lipid, then infusion of 0.25 ml/kg/minute for 60 min	After the infusion, bradycardia resolved completely. The patient was discharged in good health after 24 h of follow-up without complications.
(35)	Synthetic cannabinoid (“Bonzai”)	Male	17	Accelerated junctional rhythm	Bolus 1.5 ml/kg, 20% of lipid, then infusion of 0.25 ml/kg/minute for 60 min	At the fifth minute of infusion, the rate of ventricular extrasystole (VES) decreased. At the end of the infusion, at the sixteenth minute, the heart rate was 74/minute without VES. The patient was discharged in good health after 24 h of follow-up without complications.
(35)	Synthetic marijuana	Male	27	Hallucinations, severe psychosis, nausea, vomiting	10 mg of olanzapine orally to manage acute psychosis; prometazine to reduce nausea and vomiting	The patient was discharged after the symptoms of psychosis subsided.
(78)	25B-NBOe	Male	30	Aggression	Midazolam 15 mg lorazepam 2 mg	Three hours after administration, the patient was cooperative and oriented. He was later released from the emergency department in the presence of a family member.
(78)	25B-NBOe	Male	42	Aggression, diaphoresis, dilated pupils, muscle spasms, tremor, hyperflexia	Midazolam 12 mg	The patient was released 13 h after arriving at the emergency department.
(78)	25B-NBOMe	Male	23	Confusion, aggression, dilated pupils	Midazolam 35 mg ketamine 75 mg	Seven hours after drug administration, the patient was oriented and willing to cooperate. He did not remember using drugs.
(78)	25B-NBOMe	Male	26	Diaphoresis, salivation, high blood pressure	Midazolam 10 mg + 6 mg – the second dose within 90 min	After 90 min, the patient was calm and cooperative. Five hours after his arrival, his vital signs returned to normal.
(78)	25B-NBOMe	Male	22	Hyperthermia, increased pressure, dilated pupils	Midazolam 55 mg lorazepam orally 4 mg	The patient was willing enough to cooperate that the symptoms subsided 3 h after arriving at the emergency department.
(78)	25B-NBOMe	Male	21	Dry hot skin, dilated pupils	35 mg of midazolam within 60 min	His airway was maintained, manageable behaviour.
(78)	25B-NBOMe	Male	24	Agitation, aggression, hallucinations, irrational behaviour, self-mutilation, hyperthermia, hyperhidrosis, hypertension, nystagmus, dilated pupils	7.5 mg of intramuscular midazolam twice, 5 mg of haloperidol and midazolam in a total dose of 23.5 mg within 60 min and a combination of propofol (for 9 h), fentanyl and suxamethonium chloride	After 9 h in the ICU, propofol was discontinued. The patient was calm and willing to cooperate, so he was extubated. He did not remember the events of the previous evening. He remained under observation.
(44)	Ethylophenidate+ benzocaine (“el blanco”)	Male	>30	Accelerated relapses of schizophrenia	0.5 mg of clonazepam four times a day with haloperidol and lorazepam to control acute behavioural disorders and agitation. 10 mg of olanzapine twice daily as antipsychotic was changed to 50 mg of pipothiazine palmitate every 4 weeks	After reaching therapeutic levels of clozapine, the patient stabilised enough to engage in discussion and education about the use of NPS. He was released from the hospital 6 months after admission.
(65)	U-47700	Male	26	Cyanosis, respiratory collapse, tachycardia	As needed: oxygen therapy, ketamine, lorazepam, rocuronium bromide; chronic treatment: pulmonary ventilation, propofol	The patient was discharged after 3 days of hospitalisation after stabilisation and obtaining normal test results.
(65)	U-47700	Female	24	Drowsiness, anxiety, nausea, stomach ache	Supportive care	After 24 h of observation, the patient was discharged from the hospital.
(75)	Flubromazolam	Male	24	Loss of consciousness, fever, changes in the white matter of the brain, hypoxia brain damage, changes in EEG, permanent dilated pupils, death (due to overdose)	Intubation and oxygen therapy, from the sixth day of hospitalisation with meropenem and fluconazole (no improvement), from the 15th day with levitracetam and scopolamine with albuterol/ipratropium bromide, from the 20th day with flumazenil (no improvement)	After 19 days of hospitalisation, the patient was unresponsive and suffered from pain stimuli. On the 21st day, he was transported to the hospice where he died on the 30th day. The cause of death was complications due to overdose of psychoactive substances.
(79)	U-47700	Male	22	Loss of consciousness, apnea, gasping, cyanosis, hypoxia, difficulty thinking	Oxygen therapy, 2 mg of naloxone	No information.
(50)	MDMB-CHMICA Synthetic cannabinoid ‘'Sweat leaf”	Male	33	Epilepsy, paranoia, aggression, acidosis	10 mg of diazepam, intravenous electrolytes	The patient was discharged from the hospital after 51/52 hours at his own request.
(50)	MDMB-CHMICA Synthetic cannabinoid ‘pandora reborn'	Male	23	Hallucinations, aggression, hyperhidrosis, insomnia, self-harm, hyperactivity, tachycardia, fever, acidosis, dilated pupils	3 mg of lorazepam, intubation and ventilation, phenylephrine and noradrenaline, benzodiazepines and haloperidol	The patient remained aggressive and agitated for several consecutive days of hospitalisation as a result of which he was given high doses of benzodiazepines and haloperidol. He was discharged after 9 days without complications.
(71)	3-MeO-PCP	Male	37	Hypertension, tachycardia, psychosis, increased muscle tone with spastic leg posture	Oxygen therapy, gastric lavage with the application of activated carbon, macrogol, metoclopramide, synostigmine	The first vital signs returned 10 min after naloxone administration, itching and anxiety subsided, the patient was sleepy for 2 h, but she was able to wake up and talk coherently; she was discharged 4 h after arrival.
(53)	U-47700 Fentanyl Acetaminophen	Female	41	Reduced level of consciousness, anxiety, itching	0.4 mg of naloxone intravenously, 1 mg of lorazepam, 50 mg of diphenhydramine	The first vital signs returned 10 min after naloxone administration; itching and anxiety subsided; the patient was sleepy for 2 h, but was able to wake up and talk coherently; she was discharged 4 h after arrival.
(51)	JWH-122	Male	18	Hallucinations, blurred vision, tachycardia	6 mg of clonazepam (daily)	Visual hallucinations and visual disturbances persisted for 4 years.
(56)	Black Mamba	Female	25	Agitation, aggression	2 mg of lorazepam, 5 mg of haloperidol, 50 mg of diphenhydramine	The patient was discharged after 10 h of observation in the emergency department.
(56)	Black Mamba	Female	23	Chest pain, ECG T wave inversion, diarrhea, nausea	15 mg of ketorolac, 4 mg of ondansetron, 1 g of acetaminophen	The patient was discharged after 9 h of observation in the emergency department.
(56)	Black Mamba Marijuana	Male	27	Palpitations, bilateral numbness of the hands, nausea, vomiting	1 mg × 2 of lorazepam, isotonic NaCl 1 l solution	The patient was discharged after 3 h.
(69)	25I-NBOMe	Male	27	Agitation, aggression, blood pressure: 139/90 mm Hg, tachycardia, heart rate: 146 beats per minute (bpm), respiratory rate: 28 breaths/min *Re-hospitalisation after 3 weeks:* II. Temperature: 36.80 C, heart rate: 120 bpm, blood pressure: 153/119 mm Hg and respiratory rate: 22 breaths/min, 4 mm pupils, agitation, aggression, hallucinations	2 mg of lorazepam, 5 mg of haloperidol lactate, 2 l of 0.9% saline lorazepam at the doses of: 4 mg, 2 mg, 8 mg and 16 mg (given within 4.5 h)	I. The patient escaped from the hospital.II. The patient calmed down, vital signs improved, the patient did not require airway support, the symptoms of poisoning disappeared. On the second day of admission, the patient was already vigilant and could be assessed psychiatrically. It was recommended to move the patient to an addiction centre. However, the patient discharged himself after 48 h.
(58)	King Kong	Male	47	Fever, heart rate: 104 beats per minute, respiratory rate: 20 breaths per minute, blood pressure: 153/103 mm Hg, psychosis, delusions, agitation, hallucinations	10 mg of olanzapine, then 10 mg of haloperidol in combination with 2 mg of lorazepam and 50 mg of diphenhydramine	The administration of olanzepine did not cause sedation, the patient was still stimulated and, therefore, the other drugs were administered intramuscularly. The patient stayed in isolation overnight to reduce stimuli. The next day the patient was calm and stable.
(80)	3-MeO-PCP	Male	19	Tachypnea, tachycardia, hypertension, catatonia and mydriasis, fever, lactic acidosis, hallucinations, agitation	Diazepam, haloperidol, propofol	The patient calmed down, but saturation decreased and so he was given propofol, intubated and transferred to the ICU, where he had a high fever. After 22 h of intensive therapy, the patient completely recovered and was transferred to a psychiatric ward.
(81)	3-FPM Etizolam	Male	33	Fever: 38.9°C, coma, sinus heart rhythm, respiratory acidosis, dilated pupils, unresponsive, incorrect four limb movements	2 mg of naloxone intravenously, ketamine and succinylcholine for intubation, infusion of propofol, lorazepam, vancomycin and metronidazole	After 5 days, the patient developed new extensive T-wave inversions. After 7 days, the patient was asymptomatic.
(25)	a-PVP	Male	28	Psychosis	Risperidone (4 mg/day), venlafaxine (75 mg/day), biperiden (4 mg/day), delorazepam (2 mg/day) bupropion (150 mg/day)	Hospitalisation lasted 40 days. In addition to drug treatment, it included psychotherapy and rehabilitation. The patient's health condition improved.
(54)	Synthetic cannabinoid	Male	28	Auditory hallucinations, persecution delusions, disorganisation of thoughts, psychosis, PANSS score 109/210	37.5 mg of risperidal every 2 weeks, olanzapine 10/20 mg/day, pregabalin 100 mg/day clonazepam 8 mg/day	Positive response to the pharmacological treatment used. Symptoms decreased after 24 h, and after 7 days the PANSS score was reduced to 74/210.
(54)	Poly-substance misuse (mainly crack cocaine and heroin) Synthetic cannabinoid	Female	32	Delusions, increased aggression, excessive excitability	Aripiprazole 30 mg/day Lithium carbonate 800 mg/night haloperidol 10 mg/day clonazepam 8 mg/day	The patient's condition improved after the use of pharmacotherapy and the introduction of stricter rules of searching patients for drugs
(54)	Poly-substance misuse (benzodiazepines, synthetic cannabinoides)	Male	20	Excessive sexual arousal, aggression, thinking disorder, PANSS score 116/210	Haloperidol decanoate 50 mg/monthly Haloperidol 10 mg/night Apiprazole 9.75 mg/three times a day Clonazepam 6 mg/day	Clinical improvement after 72 h and reduction of PANSS score to 98/210.
(54)	Poly-substance misuse (benzodiazepines, synthetic cannabinoids, THC)	Male	39	Stimulation, aggression, manic episodes, hyperactivity, PANSS score: 108,123/210	Aripiprazole 400 mg/monthly (intramuscularly) Zuklupentixol 300 mg/week + Sodium valproate 1,200 mg	Positive symptoms decreased and the PANSS score dropped to 66/210.Signs of aggression and delusions disappeared. Satisfactory reaction to zuklupentixol.
(72)	3-HO-PCP N-Ethylhexedrone ‘'heksen/NEH”	Male	56	Drowsiness, diaphoresis, high blood pressure, hallucinations, hyperthermia, tachycardia	4.3 l of electrolytes, 7.5 mg of diazepam	On the second day of hospitalisation, the patient regained partial consciousness, came to full health during 9 days of hospitalisation and was discharged.
(63)	Cyclopropylfentanyl	Male	25	Coma, respiratory failure, myosis, hypothermia, repeated apnea, desaturation and sedation	s needed, administration of two doses of 0.4 mg of naloxone and oxygen therapy. Oxygen therapy and naloxone supply for another 12 h of hospitalisation.	After administration of naloxone, breathing stabilised; the patient also showed reduced oxygen saturation for the following 12 h. After 1 day of observation, he was discharged.
(70)	3-MeO-PC	Male	17	Impaired consciousness, hyperactivity, tremor, nystagmus, dysarthria, respiratory acidosis	1 mg of midazolam, 1,500 ml + 3,000 ml of NaCl solution, cyamemazine and loxapine	In <24 h, neurological disorders decreased and blood pressure and heart rate stabilised. The patient maintained moderate sedation the next day. He was transported to a psychiatric hospital, from which he was discharged after a few days.

**Figure 1:**
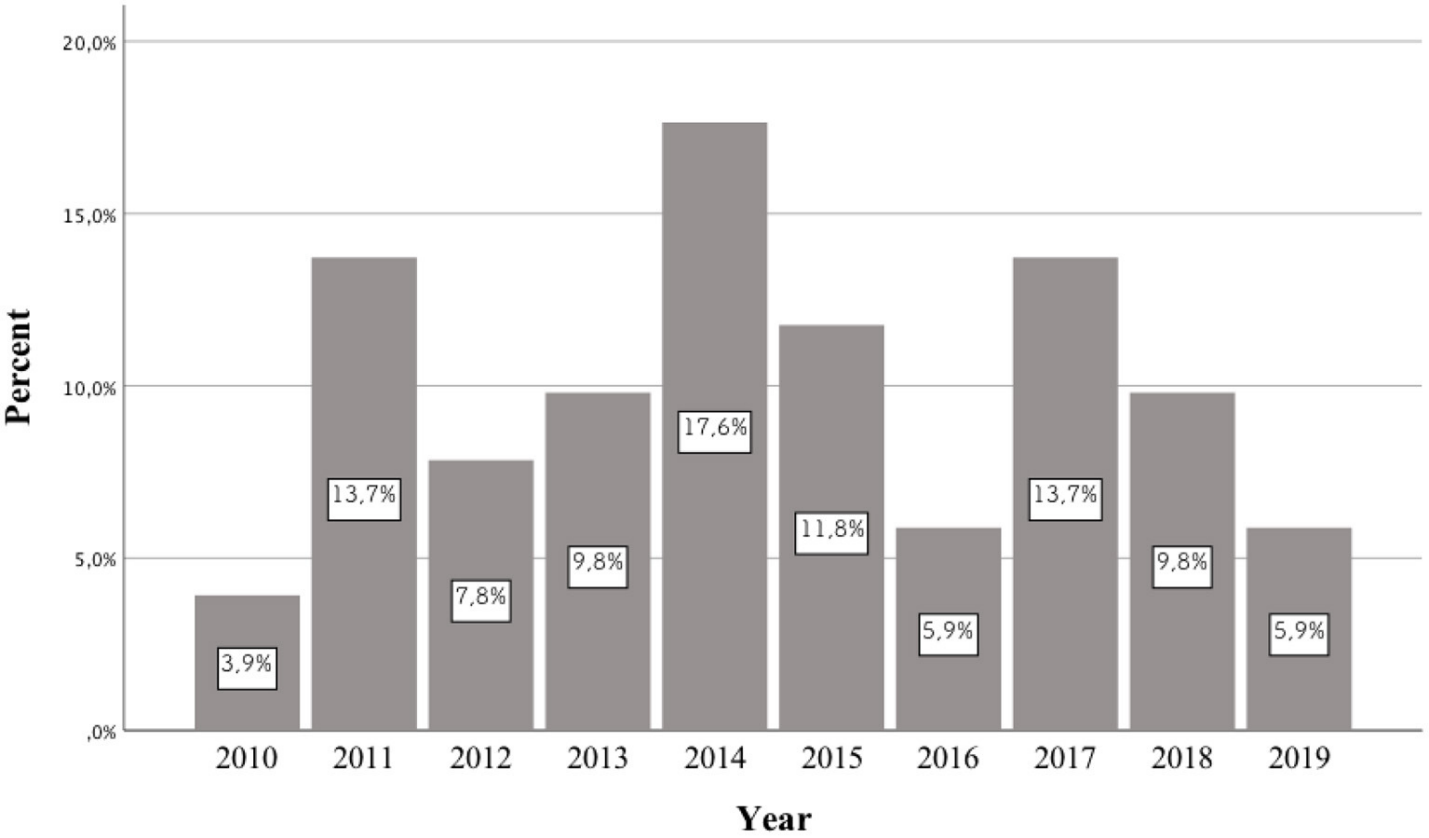
Percentage of case reports concerning patients taking NPS written between 2010 and 2019.

Most case reports were published in 2014 (Figure 1).

Men and people aged 21 to 30 were the most numerous groups. Synthetic cannabinoids and cathinones were the most commonly consumed NPS. Moreover, 62.7% of the patients described took benzodiazepines and most of them received only this group of medications, followed by those who were administered benzodiazepines combined with neuroleptics and benzodiazepines combined with muscle relaxants. The most common clinical symptoms were nervousness and confusion (Table 2).

**Table 2:** Age, gender, clinical symptoms and treatment of patients taking various types of NPS in reports written between 2010 and 2019; *chi-square.

**Variable**		***n***	**%**	Statistical test result*
Age (years)	<20	10	21.7	χ^2^(3) = 19.74; *p* < 0.001
	21–30	24	52.2	
	31–40	8	17.4	
	>40	4	8.7	
Gender	Male	43	15.7	χ^2^(1) = 24.02; *p* < 0.001
	Female	8	84.3	
New psychoactive substances	Synthetic cathinone	16	31.4	χ^2^(5) = 40.18; *p* < 0.001
	Synthetic cannabinoid	21	41.2	
	Synthetic opioid	8	15.7	
	Phencyclidine derivatives	3	5.9	
	Amphetamine-derived stimulants	2	3.9	
	Designer benzodiazepines	1	2	
Clinical symptoms	Tachycardia	17	33.3	χ^2^(1) = 5.67; *p* = 0.02
	Nervousness	22	43.1	χ^2^(1) = 0.96; *p* = 0.33
	Hallucinations	15	29.4	χ^2^(1) = 8.64; *p* = 0.003
	Seizures	11	21.6	χ^2^(1) = 16.49; *p* < 0.001
	Hyperthermia	9	17.6	χ^2^(1) = 21.35; *p* < 0.001
	Insomnia	4	7.8	χ^2^(1) = 36.26; *p* < 0.001
	Disorientation	21	41.2	χ^2^(1) = 1.59; *p* = 0.21
Treatment	Benzodiazepines	13	25.5	χ^2^(12) = 35.92; *p* < 0.001
	Neuroleptics	3	5.9	
	Benzodiazepines+neuroleptics	8	15.7	
	Opioid drugs	5	9.8	
	Benzodiazepines + muscle relaxants	6	11.8	
	Antipsychotics	3	5.9	
	Benzodiazepines + antipsychotics	3	5.9	
	Neurolepics + antipsychotics	3	5.9	
	Barbiturates	1	2.0	
	Opioid drugs + benzodiazepines	2	3.9	
	ILE bolus	2	3.9	
	Pain medications	1	2.0	
	Antidepressants	1	2.0	

The results of the logistic regression analysis concerning the occurrence of the most common clinical symptoms associated with the use of NPS, such as tachycardia, nervousness, hallucinations, seizures and disorientation are presented below. Of the individual NPS, synthetic cathinones and cannabinoids (*n* > 10 patients) were included in the analysis. Taking synthetic cathinones and, to a slightly greater extent, cannabinoids evokes nervousness in patients. It is also noteworthy that synthetic cathinones, unlike synthetic cannabinoids, have a statistically significant influence on the occurrence of confusion in patients (Table 3). Other NPS were not included in the analysis as they were characterized by a low number.

**Table 3:** Clinical symptoms of synthetic cathinone and cannabinoid intake.

**Clinical symptoms**	**Odds ratio; 95% CI**	
	**Syntethic cathinones**	**Syntethic cannabinoids**
Tachycardia	0.8; 0.18–3.46 (*p* = 0.77)	0.41; 0.1–1.79 (*p* = 0.24)
Nervousness	**7.71; 1.28–46.36 (*****p*** **=** **0.03)**	**6.6; 1.18–37.03 (*****p*** **=** **0.03)**
Hallucinations	2.5; 0.55–−11.41 (*p* = 0.23)	0.42; 0.08–2.25 (*p* = 0.31)
Seizures	0.86; 0.1–7.04 (*p* = 0.89)	3; 0.52–17.27 (*p* = 0.22)
Hyperthermia	0.83; 0.16–4.21 (*p* = 0.83)	0.13; 0.01–1.27 (*p* = 0.08)
Disorientation	**10; 1.64–60.92 (*****p*** **=** **0.01)**	4.5; 0.8–25.34 (*p* = 0.09)

*Bold values, Statistically significant results (p < 0.05)*.

### NPS and Applied Treatment

**Figure 2:**
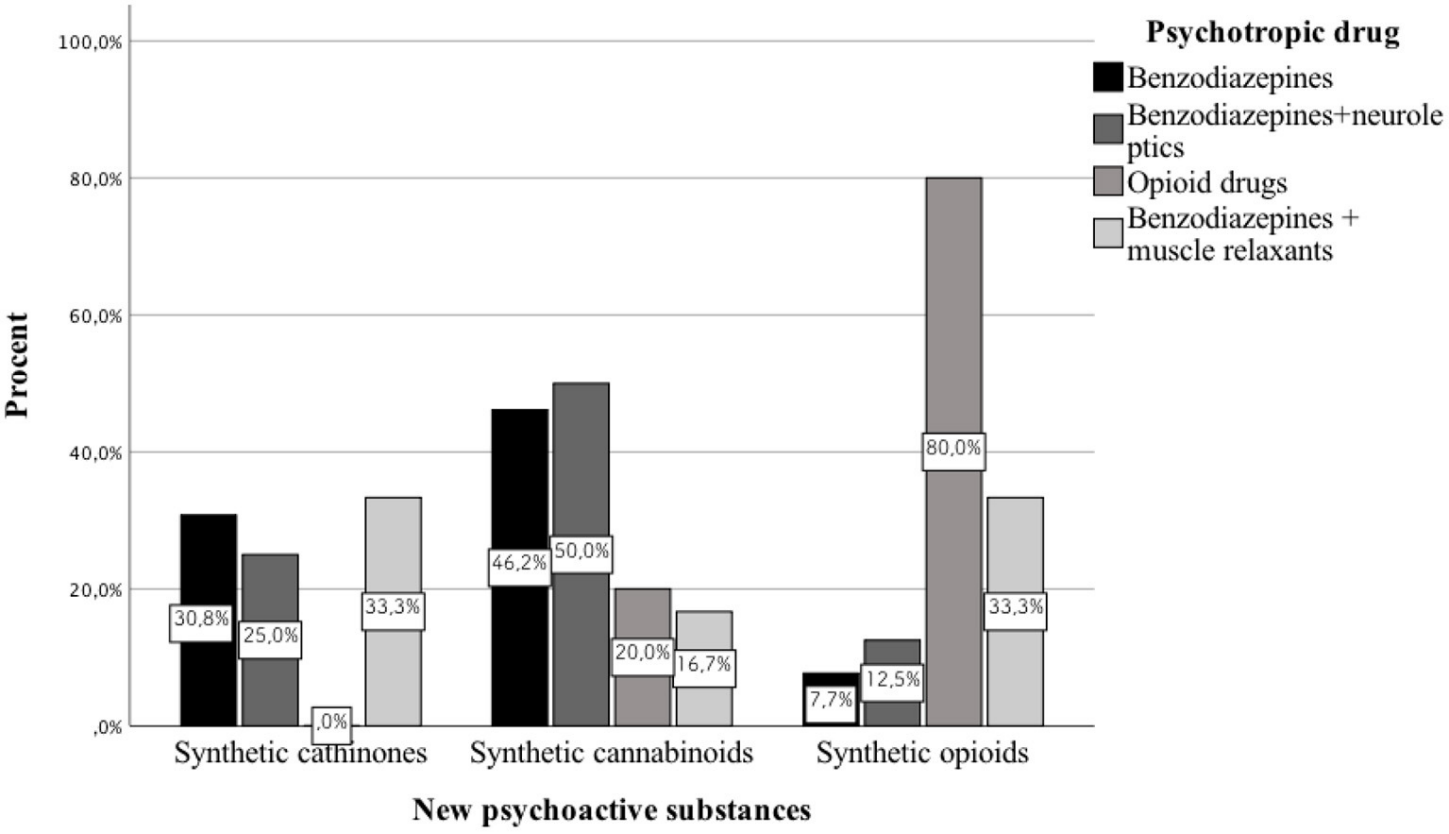
Medications used in patients taking synthetic cathinones, cannabinoids, and opioids.

Figure 2 shows the medications used in patients taking the most common NPS, that is, synthetic cathinones, cannabinoids, and opioids.

Individuals who were administered the remaining medications listed in Table 1 used mainly synthetic cathinones or cannabinoids. As for amphetamine-like stimulants, one person received benzodiazepines with opioids. One person who took phencyclidine derivatives was given benzodiazepines with antipsychotics. Moreover, benzodiazepines with muscle relaxants were prescribed to patients taking various types of NPS.

### NPS-Related Fatalities

Five of the 51 patients died. They were all men. Three of them had taken the piperazine derivative MT-45. Each of them combined MT-45 with other psychoactive substances. In the case of the first patient, this was phencyclidine and 3-methoxyphencyclidine. The second patient combined MT-45 with benzofurans, flubromazepam, pyrazolam, and alpha-pyrrolidinepentiophenone. The third man combined MT-45 with 3-methylmethcathinone and pyrazolam. Subsequent deaths in the group of patients studied concerned one patient taking 3-methylmethcathinone, 5- (2-Aminopropyl)benzofuran and alcohol, as well as one person after they consumed flubromazolam, a psychoactive substance from the designer benzodiazepines group.

In the group of patients taking MT-45, loss of consciousness, low oxygen saturation and visual impairment occurred. Psychomotor agitation, increased heart rate, convulsions and elevated temperature were observed in the patient taking 3-MMC. Death resulted when a patient took an overdose of flubromazolam. In addition to fever, the patient suffered from brain damage due to hypoxia.

It is noteworthy that three of the five patients taking MT-45 with other psychoactive substances received the opioid drug naloxone. In the patient taking 3-MMC with 5-APB and ethanol, the treatment consisted of administering diazepam, midazolam, adrenaline, and atropine. For the patient taking flubrazolam, the applied treatment consisted of administering flumazenil, as well as other medications such as meropenem, fluconazole (without improvement) and, from the 15th day of hospitalization, levitracetam and scopolamine with albuterol/ipratropium bromide.

Due to the growing problem of polypharmacotherapy in medical patients in recent years, it was decided to additionally investigate this problem in the group of 51 patients taking NPS. In this study, a statistically significant relationship was observed between the year of the published case reports on NPS intake and the total number of medications used. The later the time period, the more medications the patients were administered, *r* = −0.32; *p* = 0.02 (Odds ratio = 3.12; 95% CI 0.92–10.58). The significant relationship between the years of research and the number of drugs taken, i.e., higher than 2 is the confirmation of the obtained result, Vcramer = 0.42; *p* = 0.003.

**Figure 3:**
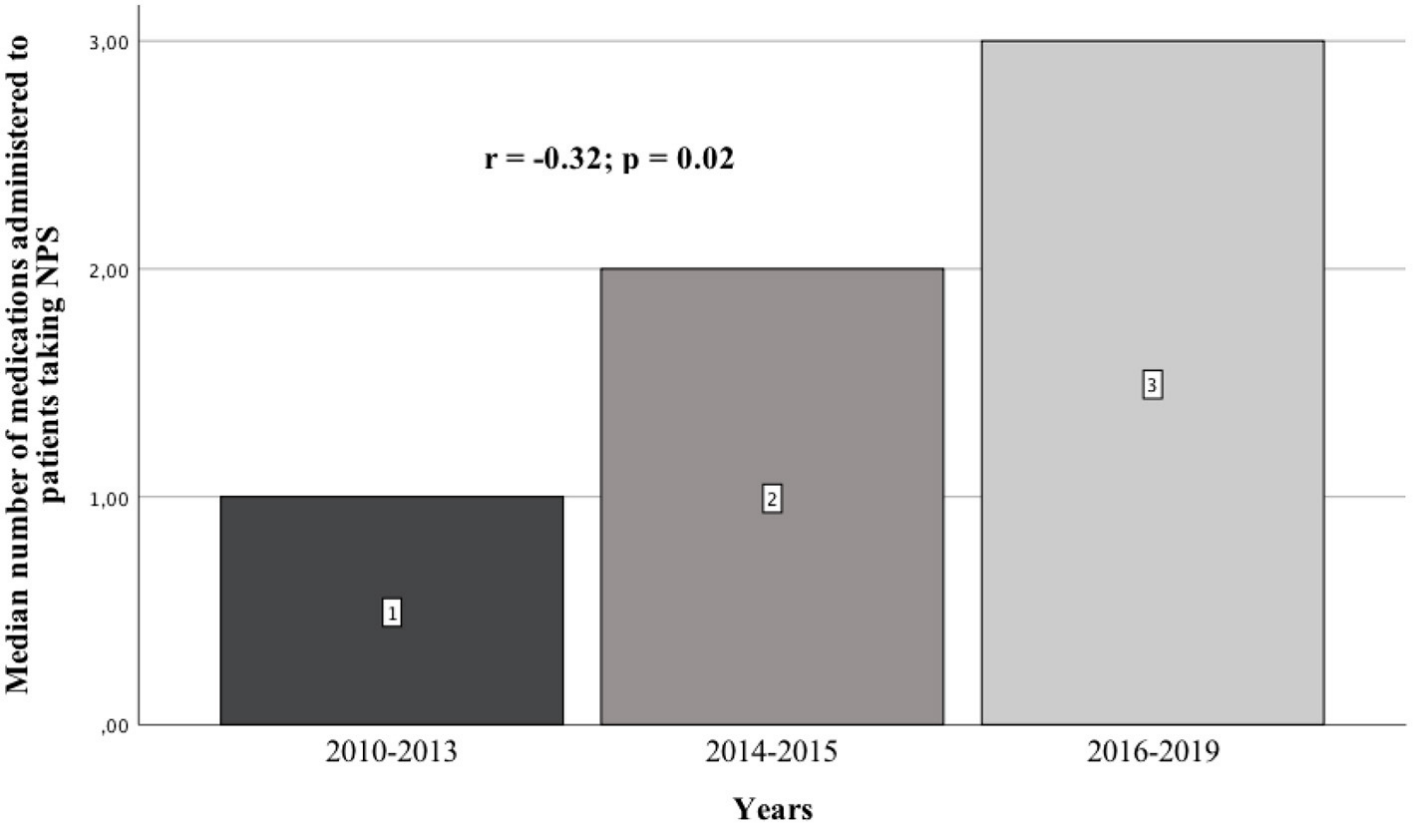
Median number of medications administered to patients taking NPS.

The number of NPS taken in total does not show a statistically significant relationship with the study period, *r* = 0.09; *p* = 0.53. With each passing time period (Figure 3), the median number of medications taken by the studied group of patients increased by one.

The result obtained is confirmed by the statistically significant relationship between the type of substance taken and the total number of medications prescribed (Table 4). With the emergence of newer and newer psychoactive substances, or in other words, as time went on, patients took more medications.

**Table 4:** Number of medications administered to patients taking various types of NPS; Me - median; *chi-square.

**New psychoactive substances**	**Number of medications administered to patients taking NPS**	Statistical test result*
	**Me**	
Synthetic cathinones	1	χ^2^(30) = 72.03; *p* < 0.001
Synthetic cannabinoids	2	
Synthetic opioids	2	
Phencyclidine derivatives	3	
Amphetamine-derived stimulants	5	
Designer benzodiazepines	7	

## Discussion

### Clinical Characteristics

Of the analyzed cases, the largest groups included men and people aged 21 to 30. This is confirmed by scientific data showing that in the general population, men, and young people use various types of NPS much more often. For example, according to the 2019 EMCDDA report, the presence of synthetic cannabinoids was found in 60% of all drug-related deaths reported in Turkey, and the majority concerned young men aged 20 to 30 (82).

The main NPS described in the case reports under analysis include synthetic cathinones and cannabinoids. Synthetic cathinones are the most important group of substances structurally related to amphetamine, methamphetamine, and MDMA. The obtained results indicate a greater influence of synthetic cathinone intake on the occurrence of aggression in comparison to cannabinoids. The presence of a pyrrolidine ring and a quaternary amine moiety increases the lipophilicity and potency of these compounds (83). An example of this is MDPV, the consumption of which has been reported to cause fatalities as well as extremely aggressive behavior (84). Stimulation of the central nervous system by synthetic cathinones may cause nervousness, hyperactivity, irritability, anxiety, and tremors, which affects the masticatory muscles. Taking cathinones may also induce symptoms of serotonin syndrome with disorders of the central nervous system, autonomic nervous system instability and neuromuscular hyperactivity. This risk increases with the use of additional psychoactive substances, such as cocaine and amphetamine (85–87). The article has been published recently in which the authors maintain that Serotonin Syndrome (SS) may be related to over-activation of the serotoninergic system produced by several mechanisms resulting in a classic triad of altered mental status, neuromuscular effects, and autonomic hyperactivity. These substances may include: psychedelic phenethylamines and synthetic cathinones (88).

It should be remembered that patients may be aggressive toward the people around them, as well as posing a threat to themselves. The conducted analysis also shows that synthetic cathinones, unlike cannabinoids, have a statistically significant influence on the occurrence of confusion. According to the literature, cathinones contribute to the occurrence of cognitive disorders, such as confusion and long-term impairment of mental performance, as well as mental disorders such as panic attacks, aggression, often accompanied by violence, depression, and suicidal thoughts and acts (89).

The case reports regarding the intake of ever newer psychoactive substances relate to the last few years. Among other things, new synthetic opioids with a different chemical structure to opioid medications used for therapeutic purposes have appeared on the market. They pose a very serious threat to public health (36, 90). In the United States, for example, there have been a particularly high number of deaths due to opioid overdoses in recent years (91). These substances cause a euphoric effect as they bind to the presynaptic μ-opioid receptors (83). One of the main symptoms in the case reports studied was impaired thinking and a decreased level of consciousness. For example, reports on poisoning with U-47700 describe symptoms similar to those in poisoning with traditional opioids (92). Three patients taking MT-45 died as reported in the last part of the discussion. As for the remaining single published case reports, these relate to phencyclidine derivatives, amphetamine-derived stimulants and synthetic benzodiazepines. In addition to psychomotor agitation, patients taking phencyclidine derivatives had dilated pupils and nystagmus. Hallucinogenic sleep symptoms, the so-called closed-eye hallucinations are recognized as a characteristic symptom after taking methoxetamine (93). However, more research is needed to investigate the effects of these substances on the human body, their toxicokinetics and toxicity.

### Pharmacotherapy of Patients Taking NPS

The major part of pharmacotherapy used in the 51 patients taking various types of NPS consisted of benzodiazepines, and aggression was the most common symptom. The greatest number of authors described patients administered benzodiazepines alone, without any other medications. These mainly include diazepam, lorazepam, and midazolam. In the remaining patients, benzodiazepines were combined primarily with neuroleptics and muscle relaxants. Haloperidol is of the most common neuroleptics, while ocuronium and vecuronium are the most prevalent muscle relaxants.

The reduction of agitation is an essential element, *inter alia*, in the treatment of hyperthermia and high blood pressure in people poisoned with NPS. The results obtained are confirmed in the literature, indicating that benzodiazepines are the medications of choice used to control agitation and aggression. It is recommended using this group of medications in increasingly higher doses. This is related to the patient's safety, interactions between the pharmaceuticals used and the psychoactive substances taken, and the possibility of a quick reversal of the medications' effects (9). However, it should be remembered that the administration of higher doses of benzodiazepines may worsen the symptoms of already existing respiratory failure. It is important to be prepared for mechanical ventilation and endotracheal intubation while sedating (94, 95). In addition to the fact that benzodiazepines have sedative, anxiolytic and hypnotic effects, they also act as anticonvulsants (96). This is important as seizures are a common symptom in patients taking NPS.

The analysis also shows that the total number of medications prescribed to patients taking NPS increases over time. This is confirmed by the latest literature reports. The research carried out in 2020 shows that with the increase in the number of medications administered in those patients taking mephedrone (for a minimum of 2 days), the frequency of hospitalization also grows. The number of additional substances taken with NPS does not correlate with the frequency of hospitalization (3). The increase in the number of simultaneously prescribed medications may also be related to the emergence of newer, unexplored NPS, which, when taken along with other psychoactive substances, intensify the resulting clinical symptoms. The number of hospitalizations of patients who chronically take various types of newer psychoactive substances, which grows from year to year, and the related need to treat these patients with many medications at the same time, increases the risk of pharmacological interactions not only between administered medications, but also between uncontrolled addictive substances. This often makes it difficult to achieve a therapeutic effect, is one of the factors that increases the risk of re-hospitalization, and also increases the costs of treatment. One of the recommendations that may reduce the risk of re-hospitalization is to supplement patients with liver regenerating preparations. This is because both psychotropic substances taken and a series of various medications may intensify the hepatotoxic effects, and thus make it difficult to achieve a therapeutic effect (97, 98).

### NPS-Related Fatalities

Of the cases of deaths from NPS reported in the literature, three relate specifically to the synthetic opioid MT-45. The Council Implementing Decision (European Union – EU) of 2015 stated that one member state recorded a total of 28 deaths as a result of taking MT-45 between November 2013 and July 2014. The country also recorded about 18 cases of serious poisoning, the clinical features of which were similar to those of opiate intoxication. Single animal studies show that the acute toxicity of MT-45 is several times higher than that of morphine (19). Based on the information currently available, MT-45 is not widely used. The substance appears to be mainly used at home by users who want to try any new substance or are addicted to opioids and have no access to heroin or other opioids. Users combine MT-45 with other psychoactive substances (38, 99). The case reports confirm this fact. Three men combined MT-45 with other psychoactive substances. Probably for this reason, they died despite treatment with naloxone. According to the literature data, combining new synthetic opioids with other substances is considered the main risk factor for death related to their use. Clinical symptoms in patients could result from the action of the nitrogen mustards used in MT-45 synthesis, as well as the stimulation of the κ and δ-opioid receptors. The available scientific data on MT-45 are limited and it has been indicated that there is a need for further research to determine the health and social risks of this substance. In the case of many different synthetic opioids, the difference between the euphoric dose and the dose that induces central nervous system depression is very small, as a result of which tolerance develops more rapidly and can cause death (90, 100).

Another reported death case concerns a patient who overdosed on flubromazolam, a new psychoactive substance from the designer benzodiazepines group. The 2017 report of the European Monitoring Center for Drugs and Drug Addiction indicates an increasing number of poisonings with new benzodiazepines (101). This type of NPS group has an inhibitory effect on the activity of the central nervous system, resulting in hypnotic, anxiolytic and amnestic effects. This is due to the antagonistic effect on the GABA_A_ receptor. According to the literature data, clinical symptoms caused by taking designer benzodiazepines appear very quickly and are much more intense compared to classic medications. Despite the use of flumazenil with other medications, the resulting brain damage due to hypoxia contributed to the patient's death (73, 102, 103).

Another death concerned a patient taking 3-MMC with 5-APB and ethanol. Benzofurans of the 5-APB and 6-APB types cause MDMA-like effects, such as euphoria, stimulation and a sense of being at one with the world. However, these symptoms last for up to three times longer (104). These types of NPS are potent inhibitors of norepinephrine, dopamine and serotonin reuptake. Animal studies have shown that 5-APB and 6-APB are potent agonists of 5-HT2B receptors (105, 106). However, the frequency of use of this NPS group is very low. Despite benzodiazepine treatment, a patient's death could occur as a result of more severe symptoms of 5-APB poisoning when combined with 3-MMC and ethanol. In a study carried out in a group of 57 patients reporting the consumption of benzofurans alone, the following symptoms were more frequent: tachycardia, hypertension, pupil dilation, palpitations, fever, increased sweating, tremors, as well as mental health disorders (107). Due to the serotonin agonism of benzofuran, the chronic use of this compound may also be associated with heart valve disease (104).

### Limitations

In published articles it lacks of the quantitative determination of new psychoactive substances and the relationship of their concentration with the clinical condition of patients. The reason for this is that quantitative methods for the determination of new psychoactive substances have only been developed in recent years, and the problem of hospitalization of patients who abuse such substances began much earlier. Secondly, another limitation of these analyses is the fact, that they are based on the different case reports and not controlled studies. This is preliminary research referring to a number of the analyzed parameters such as: clinical symptoms, type of NPS taken and applied treatment. There is need for further research on a bigger group of people taking different NPS.

## Conclusion

Year on year, there are more and more case reports of patients taking ever newer psychoactive substances. Controlling agitation is the first step in treating this patient group. One should strive to reduce the number of medications simultaneously prescribed to patients taking NPS, as this may make it difficult to achieve a therapeutic effect.

## Data Availability Statement

The original contributions presented in the study are included in the article/supplementary material, further inquiries can be directed to the corresponding author/s.

## Author Contributions

MO: idea, writing, statistical analysis, and approval the final version of manuscript. AZ, MB, MT, KW, AK, NG, MaZ, MiZ, and EM: writing the manuscript and approval the final version of manuscript. TN: clinical aspect of psychopharmacology and approval the final version of manuscript. MB-Z: approval the final version of manuscript. All authors contributed to the article and approved the submitted version.

## Conflict of Interest

The authors declare that the research was conducted in the absence of any commercial or financial relationships that could be construed as a potential conflict of interest.

## Abbreviations

AB-PINACA: N-[1-(aminocarbonyl)-2-methylpropyl]-1-pentyl-1H-indazole-3-carboxamide
ADHD: Attention deficit hyperactivity disorder
AOT: opioid agonists
5-APB: 5-(2-aminopropyl)benzofuran
AM2201: (1-(5-fluoropentyl)-3-(1-naphthoyl)indole)
AM-694: (1-(5-fluoropentyl)-3-(2-iodobenzoyl)indole)
AM-2233: 1-[(N-methylpiperidin-2-yl)methyl]-3-(2-iodobenzoyl)indole
BB-22: 1-(cyclohexylmethyl)-1H-indole-3-carboxylic acid 8-quinolinyl ester)
25B-NBOMe: novel N-2-methoxybenzyl-phenethylamine
CPK: creatine phosphokinase
DOC: 2,5-dimethoxy-4-chloroamphetamine
DSM: Diagnostic and Statistical Manual of Mental Disorders
EMCDDA: European Monitoring Centre for Drugs and Drug Addiction
EDDP: (2-ethylidene-1,5-dimethyl-3,3-diphenylpyrrolidine)
5FADP-PINACA: N-[1-(aminocarbonyl)-2,2-dimethylpropyl]-1-(5-fluoropentyl)-1H-indazole-3-carboxamide
5F-PB-22: (quinolin-8-yl 1-(5-fluoropentyl)-1H-indole-3-carboxylate)
3-FPM: 3- uorophenmetrazine
HAT: Heroin-assisted Therapy
3-HO-PCP: 3-Hydroxyphencyclidine
ICU: intensive care unit
ILE: lipid emulsion therapy
JWH018: naphthalen-1-yl-(1-pentylindol-3-yl) methanone
JWH-073: naphthalen-1-yl-(1-butylindol-3-yl) methanone
JWH-122: (4-Methyl-1-naphthalenyl)(1-pentyl-1H-indol-3-yl)-methanone
K2: synthetic cannabinoid (Spice)
MDMA: 3,4-Methylenedioxymethamphetamine
MDMB-CHIMICA: N-[[1-(cyclohexylmethyl)-1H-indol-3-yl]carbonyl]-3-methyl-L-valine, methyl ester
MDMB-PINACA: 2-{[1-(5- uoropentyl)-1H-indazole- 3-carbonyl]amino}-3,3-dimethylbutanoate
MDPV: methylenedioxypyrovalerone
3-MeO-PCP: 3-Methoxyphencyclidine
3-MMC: 3-Methylmethcathinone
4-MMC: 4-methylmethcathinone
MT-45: 1-cyclohexyl-4-(1,2-diphenylethyl) piperazine
NPS: new psychoactive substances
NWS: neonatal withdrawal syndrome
PASS: Postural Assessment Scale for Stroke
PB22: (1-pentyl-8-quinolinyl ester-1H-indole-3-carboxylic acid)
PCP: phencyclidine
PVCs: premature ventricular contractions
PVP: alpha-pyrrolidinovalerophenone
THC: tetrahydrocannabinol
U-47700: 3,4-dichloro-N-((1S,2S)-2-(dimethylamino)cyclohexyl)-N-methylbenzamide
VES: ventricular extrasystoles.
